# Supplementary figures and images for: Hydrodynamic performance of Ordovician archaeostracan carapaces
Source: PLoS One. 2024 May 31;19(5):e0304559. doi: 10.1371/journal.pone.0304559 (PMC11142683; doi:10.1371/journal.pone.0304559)

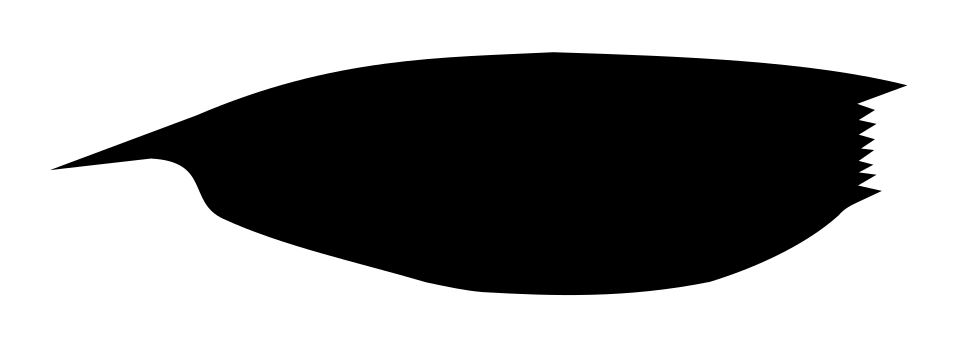

Supplement: S4 File — (ZIP) [file pone.0304559.s004.zip › 11_Caryocaris_acoitensis.JPG]

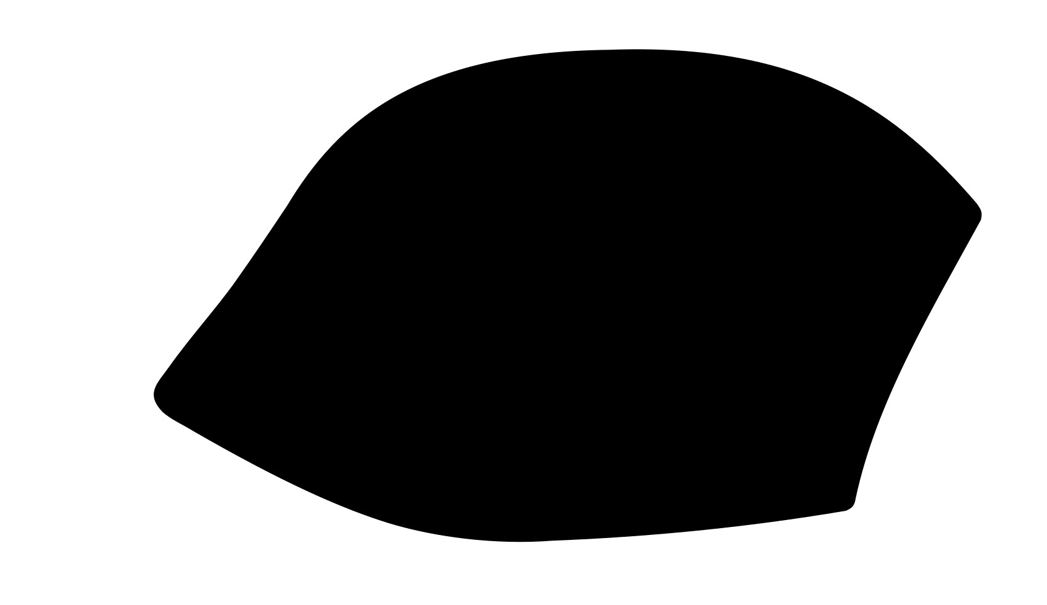

Supplement: S4 File — (ZIP) [file pone.0304559.s004.zip › 22_Ceratiocaris_papilio.jpg]

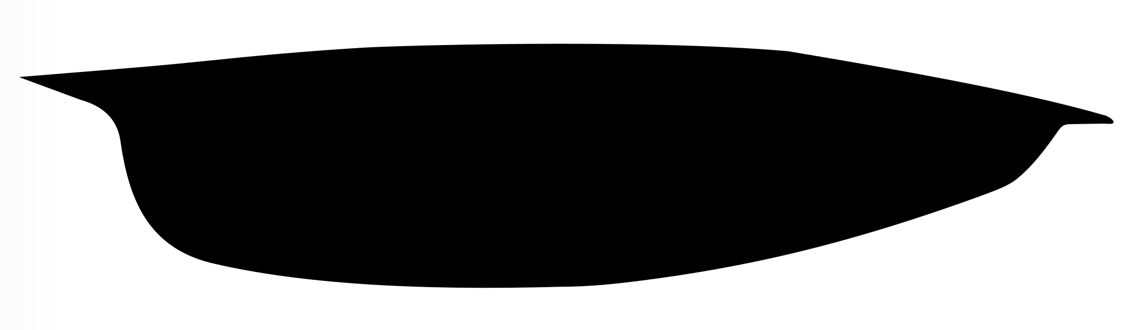

Supplement: S4 File — (ZIP) [file pone.0304559.s004.zip › 31_Ivocaris_delicata.jpg]

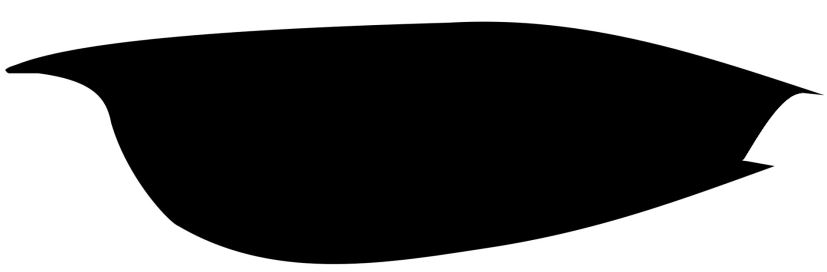

Supplement: S4 File — (ZIP) [file pone.0304559.s004.zip › 32_Ivocaris_saltitensis.JPG]

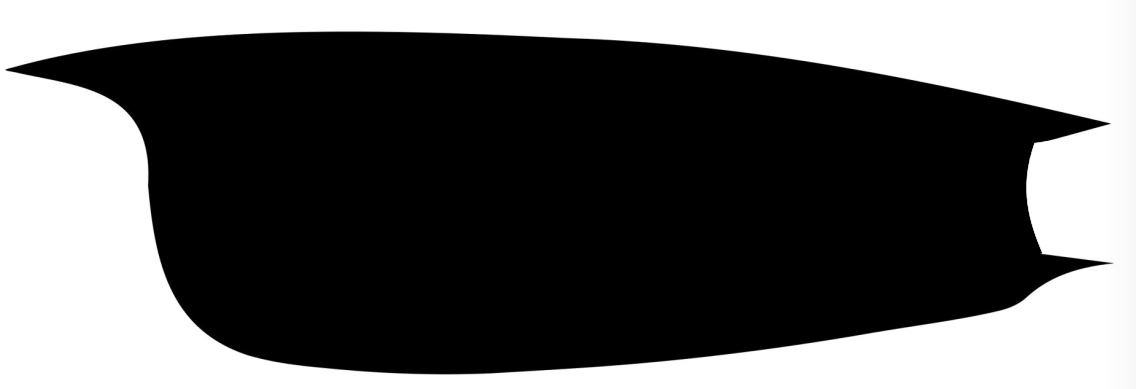

Supplement: S4 File — (ZIP) [file pone.0304559.s004.zip › 41_Janviericaris_formosa.JPG]

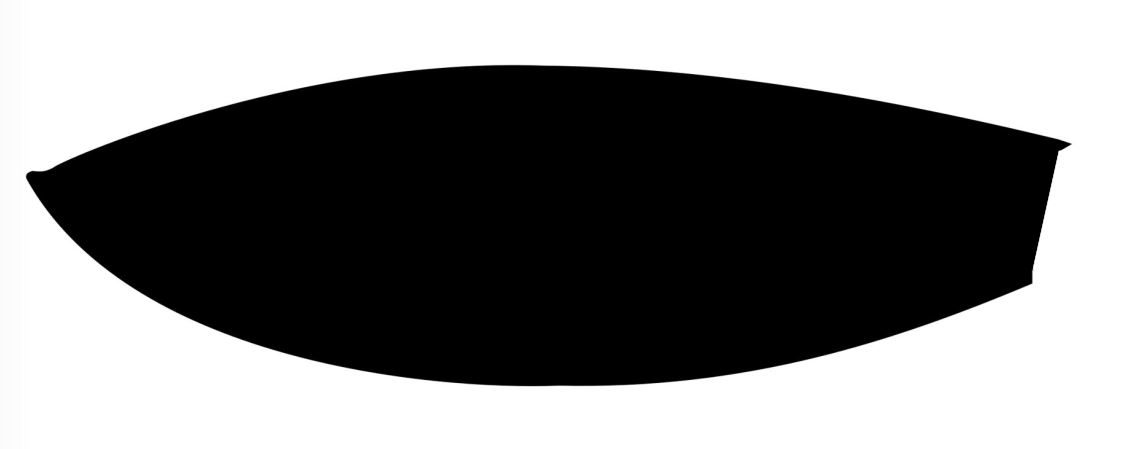

Supplement: S4 File — (ZIP) [file pone.0304559.s004.zip › 42_Janviericaris_raymondi.JPG]

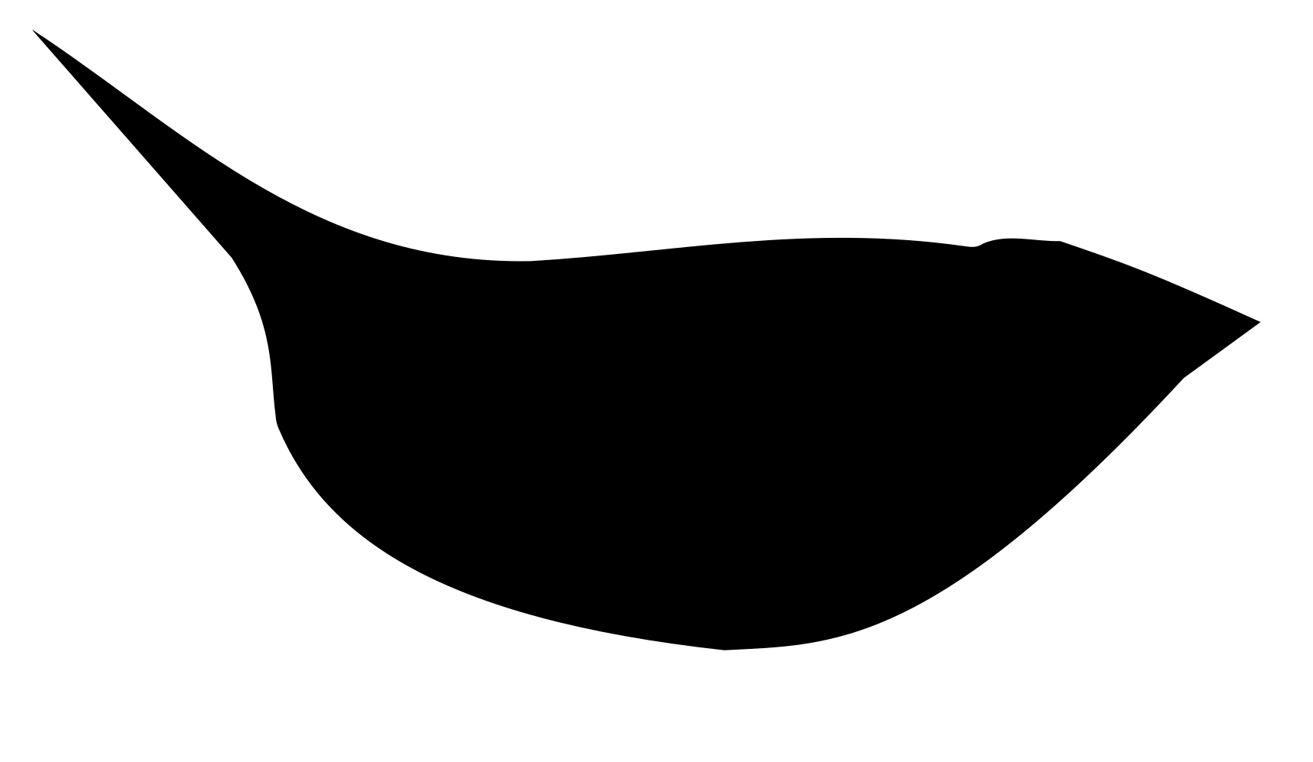

Supplement: S4 File — (ZIP) [file pone.0304559.s004.zip › 82_Soomicaris_scanicus.jpg]

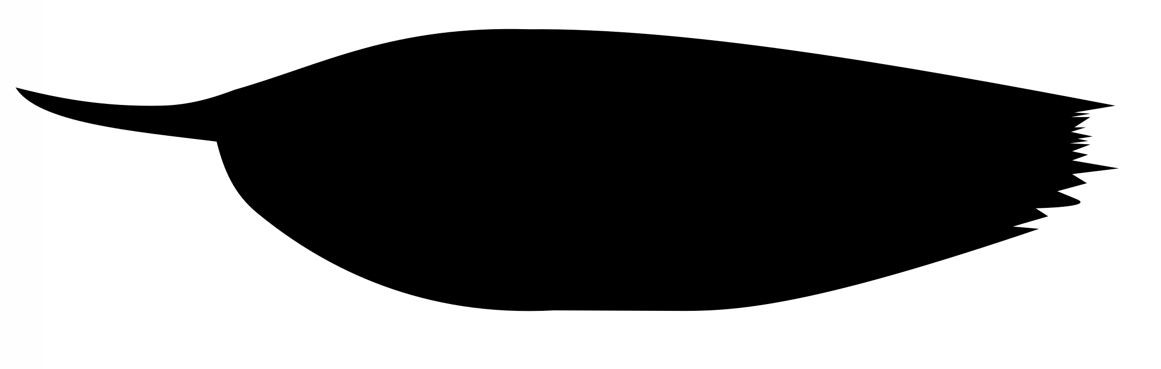

Supplement: S4 File — (ZIP) [file pone.0304559.s004.zip › 14_Caryocaris_zhejiangensis.jpg]

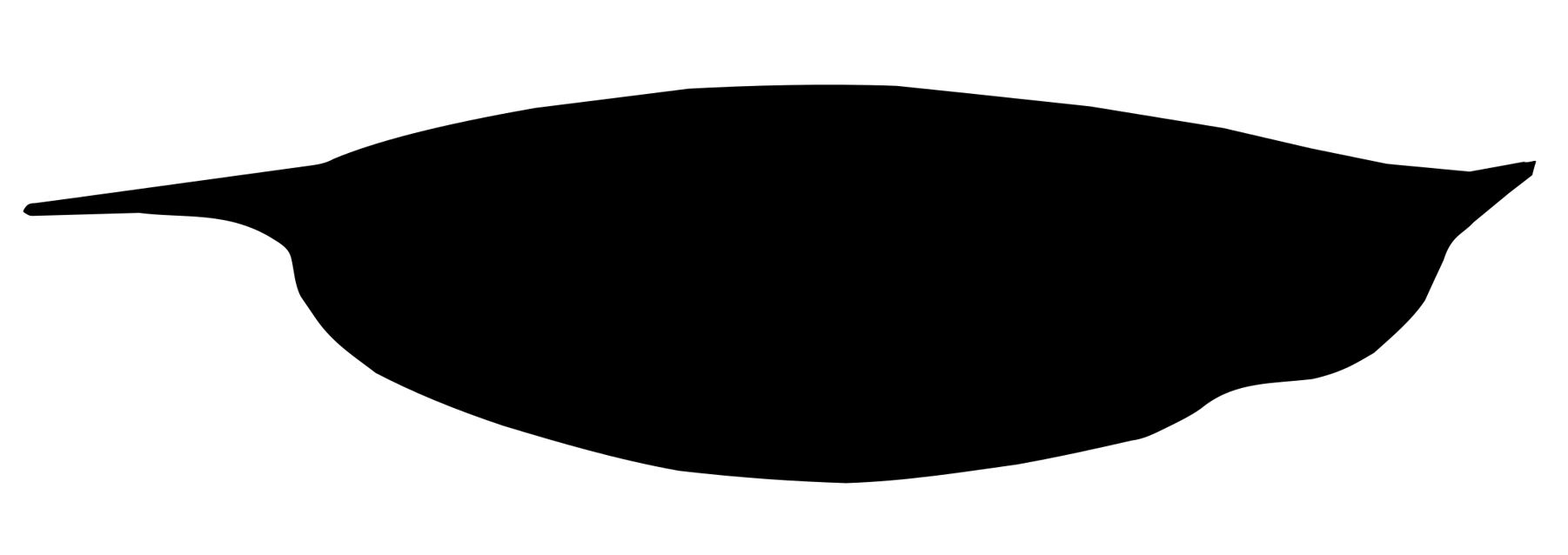

Supplement: S4 File — (ZIP) [file pone.0304559.s004.zip › 71_Saltericaris_subula.jpg]

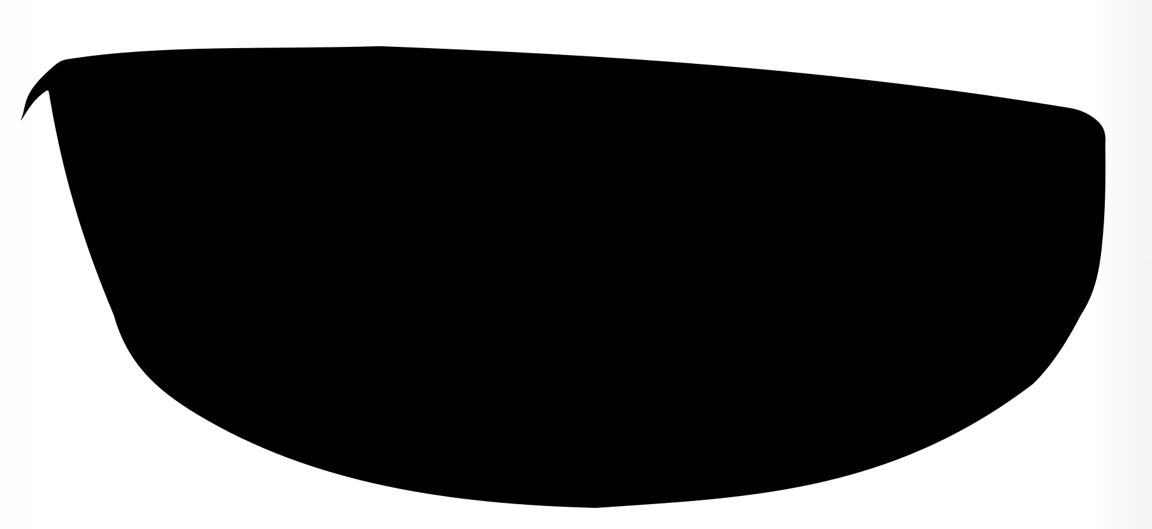

Supplement: S4 File — (ZIP) [file pone.0304559.s004.zip › 23_Ceratiocaris_pusila.jpg]

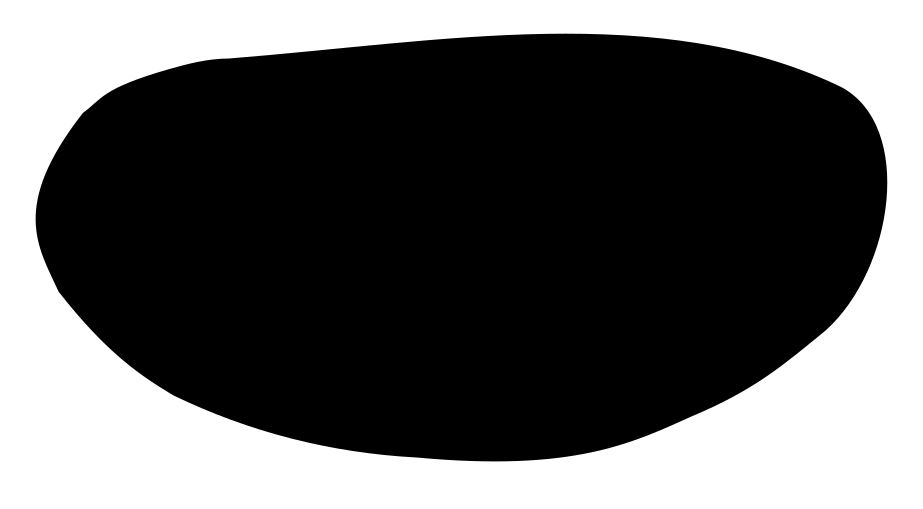

Supplement: S4 File — (ZIP) [file pone.0304559.s004.zip › 91_Rolfecaris_lethiersi.JPG]

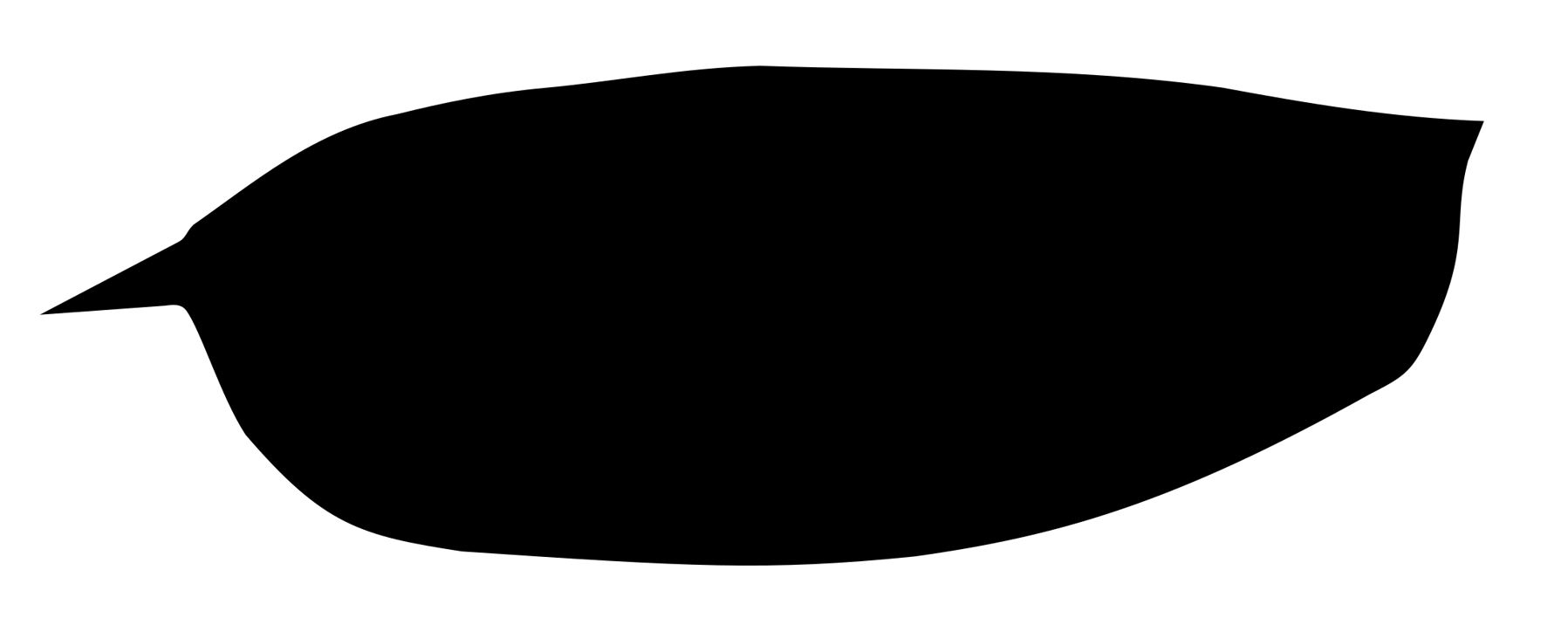

Supplement: S4 File — (ZIP) [file pone.0304559.s004.zip › 81_Soomicaris_cedarbergensis.jpg]

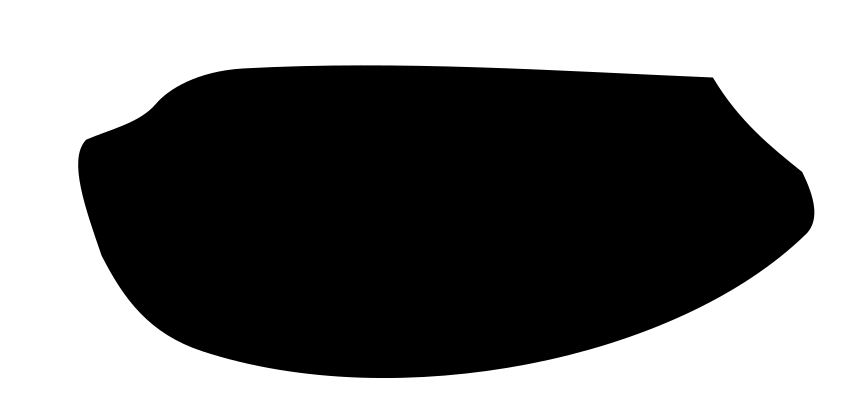

Supplement: S4 File — (ZIP) [file pone.0304559.s004.zip › 64_Pumilocaris_granulosa.JPG]

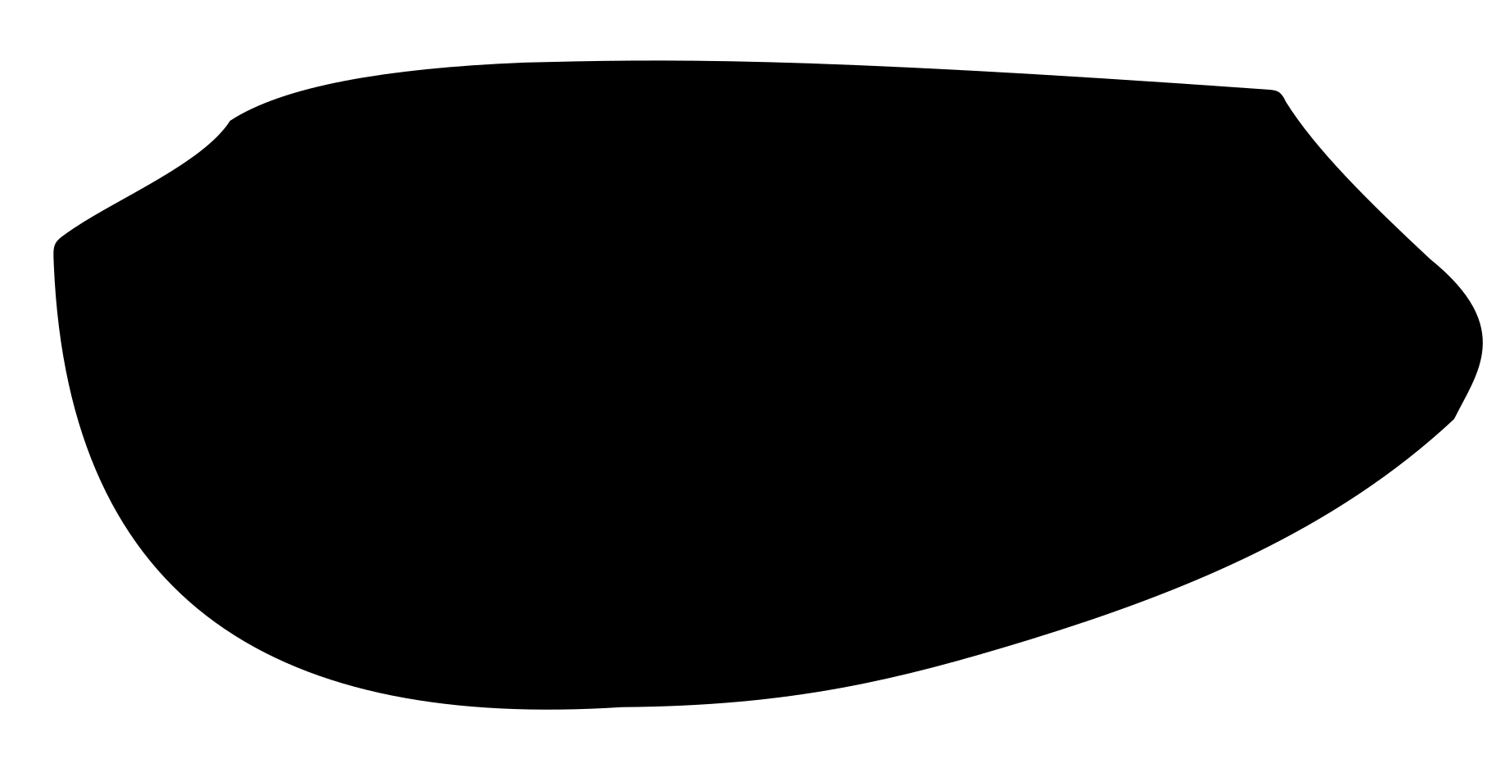

Supplement: S4 File — (ZIP) [file pone.0304559.s004.zip › 62_Pumilocaris_granulosus.jpg]

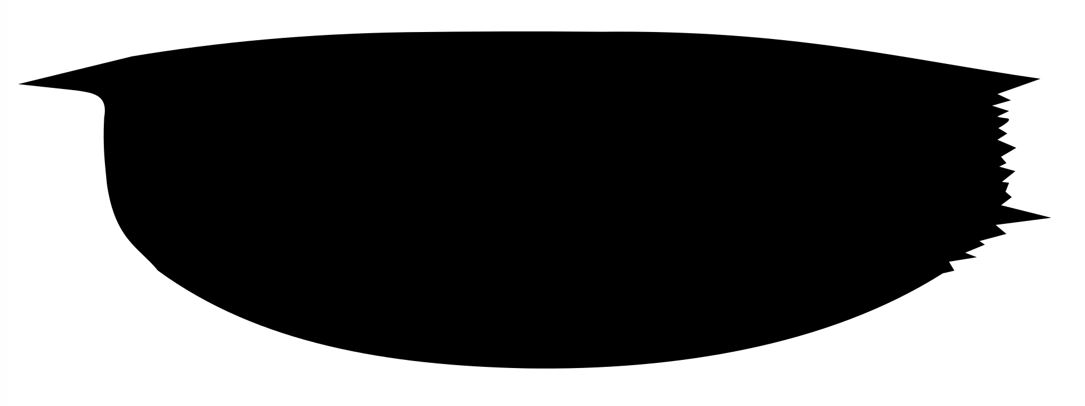

Supplement: S4 File — (ZIP) [file pone.0304559.s004.zip › 12_Caryocaris_curvilata.JPG]

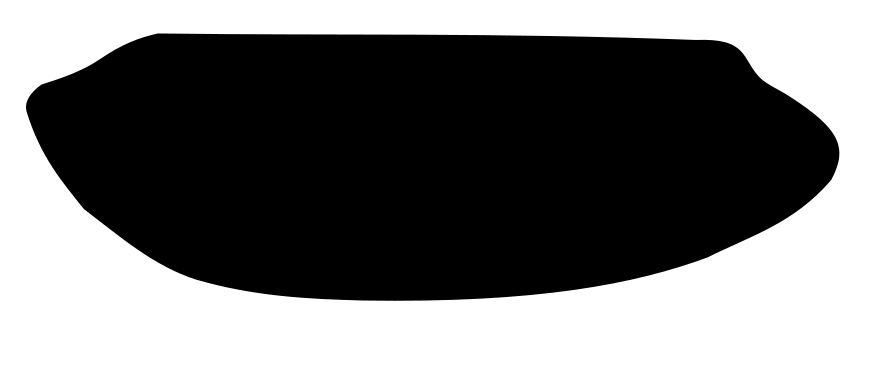

Supplement: S4 File — (ZIP) [file pone.0304559.s004.zip › 63_Pumilocaris_salina.JPG]

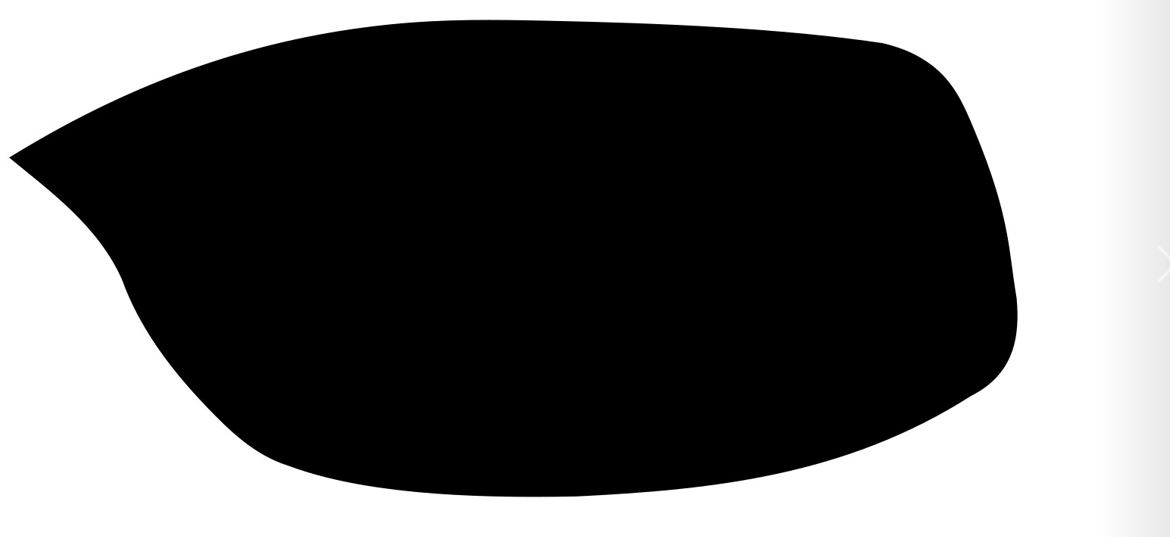

Supplement: S4 File — (ZIP) [file pone.0304559.s004.zip › 21_Ceratiocaris_macroura.jpg]

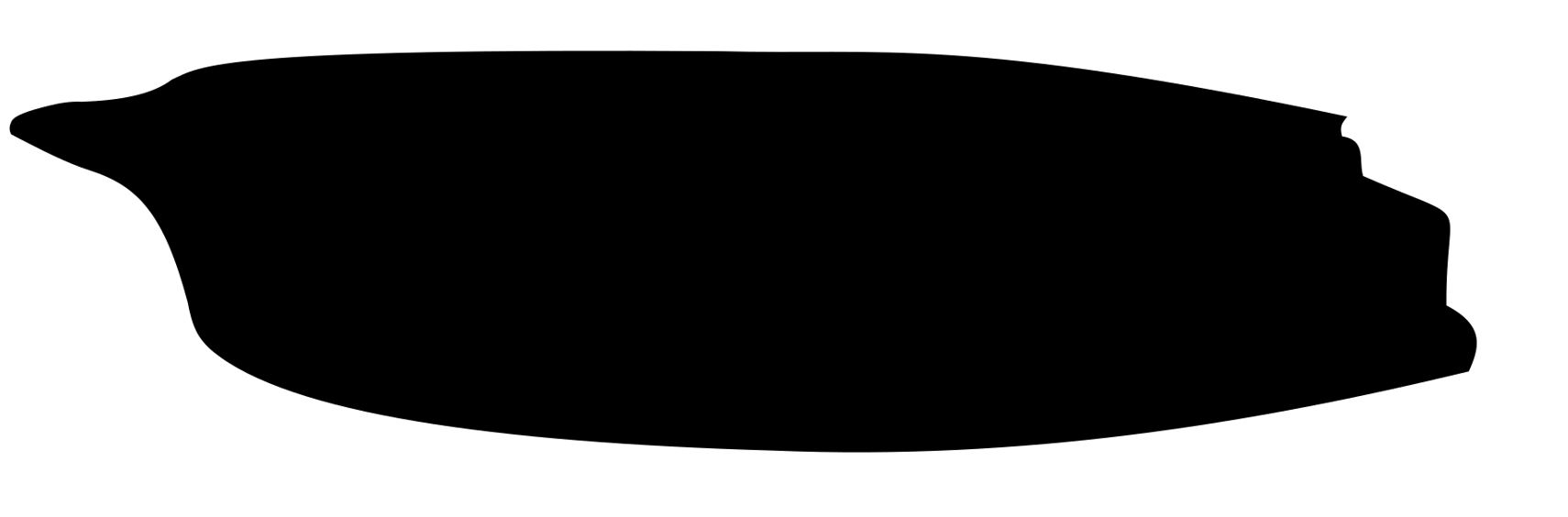

Supplement: S4 File — (ZIP) [file pone.0304559.s004.zip › 61_Pumilocaris_acuta.jpg]

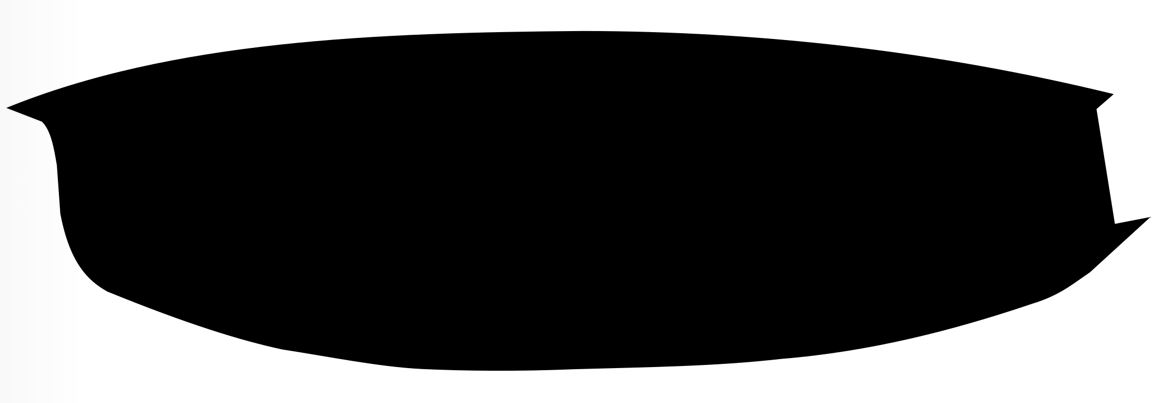

Supplement: S4 File — (ZIP) [file pone.0304559.s004.zip › 13_Caryocaris_wrightii.jpg]

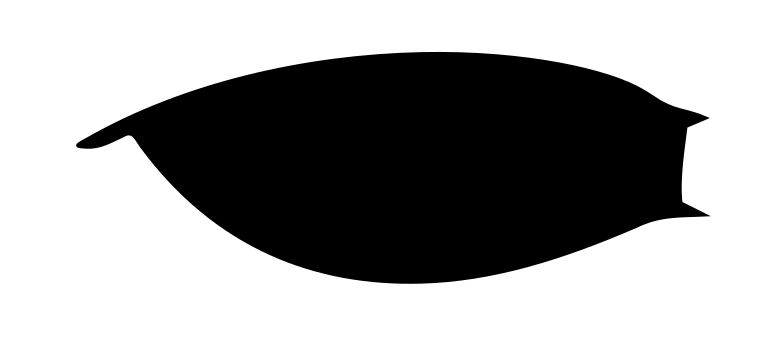

Supplement: S4 File — (ZIP) [file pone.0304559.s004.zip › 51_Jellicaris_stewarti.JPG]

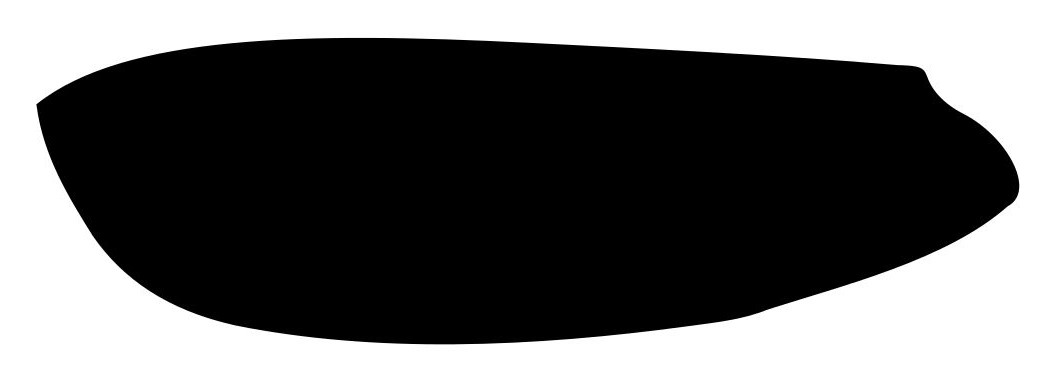

Supplement: S4 File — (ZIP) [file pone.0304559.s004.zip › 25_Ceratiocaris_silicula.JPG]

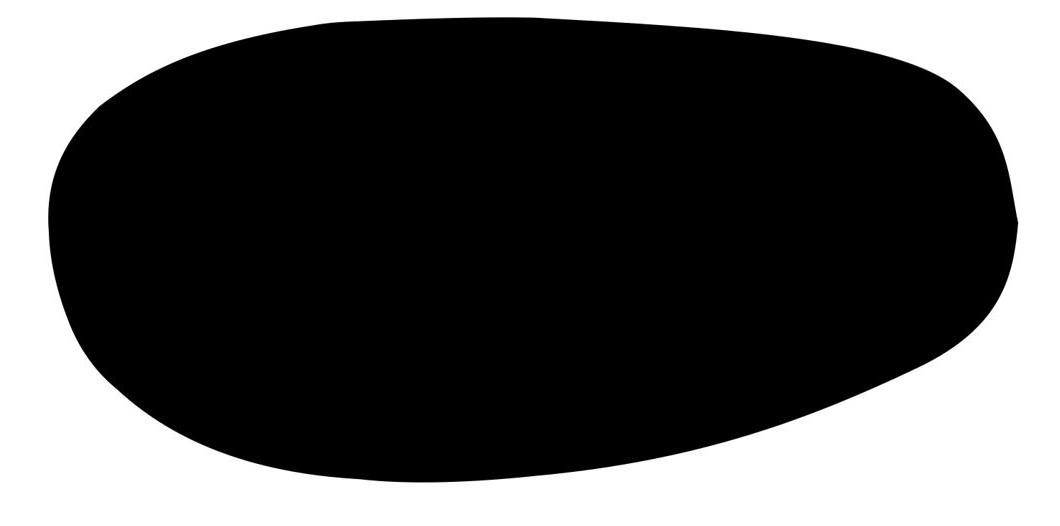

Supplement: S4 File — (ZIP) [file pone.0304559.s004.zip › 01_Arenosicaris_inflata.JPG]

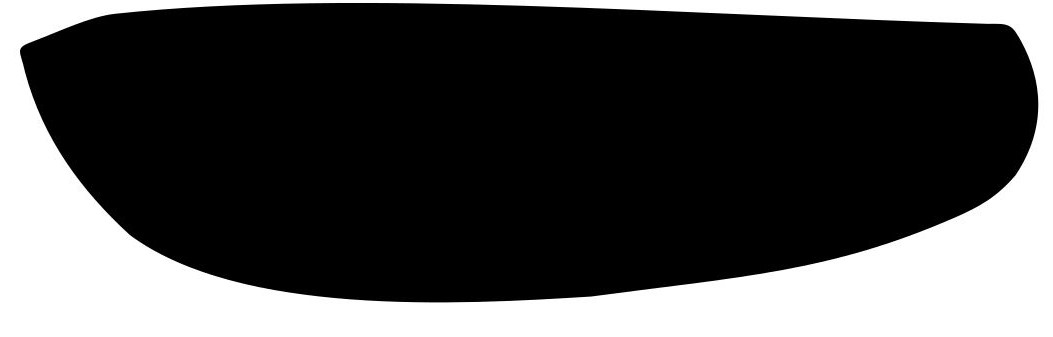

Supplement: S4 File — (ZIP) [file pone.0304559.s004.zip › 24_Ceratiocaris_angusta.JPG]

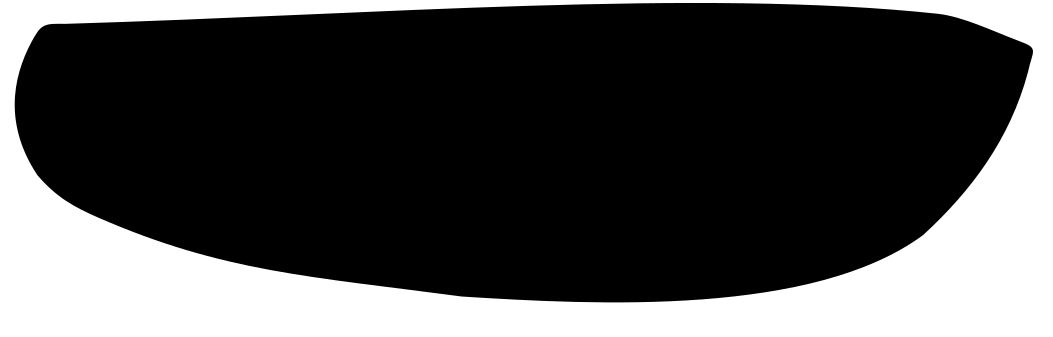

Supplement: S4 File — (ZIP) [file pone.0304559.s004.zip › V1_outlines/24_Ceratiocaris_angusta.JPG]

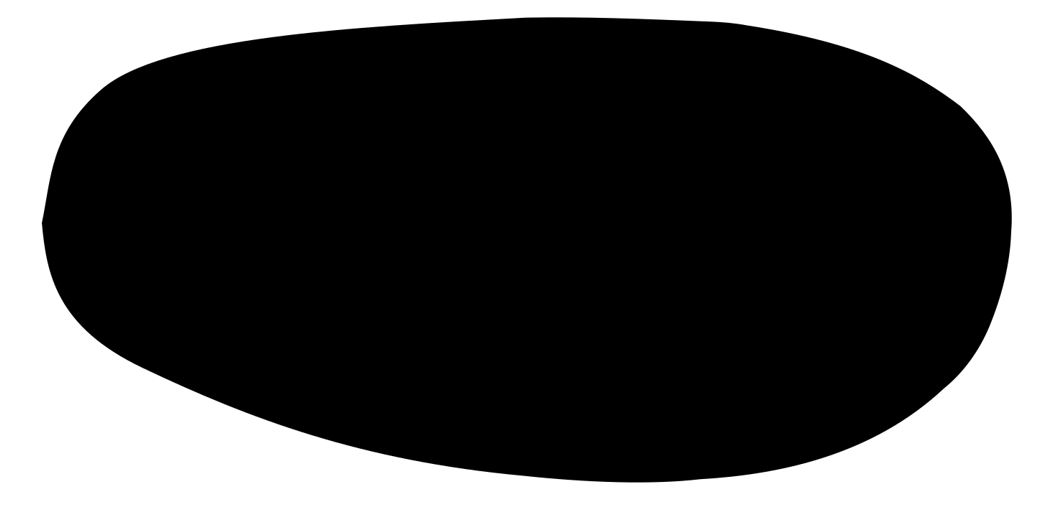

Supplement: S4 File — (ZIP) [file pone.0304559.s004.zip › V1_outlines/01_Arenosicaris_inflata.JPG]

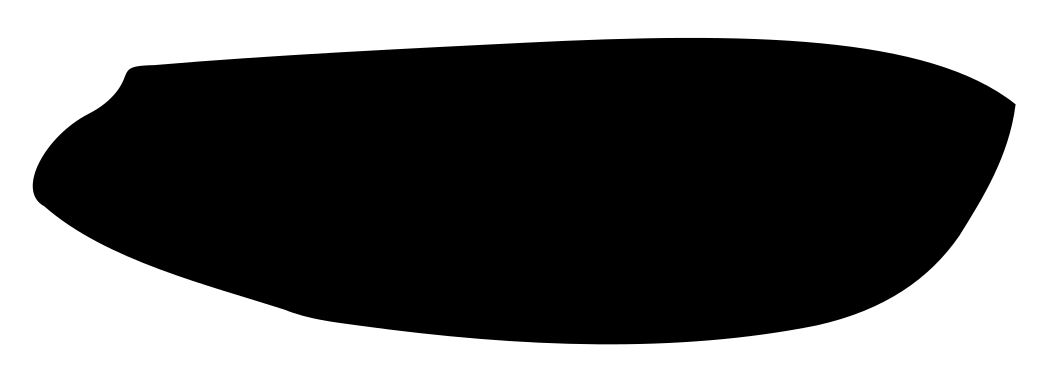

Supplement: S4 File — (ZIP) [file pone.0304559.s004.zip › V1_outlines/25_Ceratiocaris_cilicula.JPG]
