# Supplementary material for: Hydrodynamic performance of Ordovician archaeostracan carapaces
Source: PLoS One. 2024 May 31;19(5):e0304559. doi: 10.1371/journal.pone.0304559 (PMC11142683; doi:10.1371/journal.pone.0304559)
Supplement: Attachment — Submitted filename: PONE-D-23-26410.pdf [file pone.0304559.s005.pdf]

# PLOS ONE

## Hydrodynamic performance of early Palaeozoic archaeostracan carapaces

--Manuscript Draft--

| | |
|---|---|
| **Manuscript Number:** | PONE-D-23-26410 |
| **Article Type:** | Research Article |
| **Full Title:** | Hydrodynamic performance of early Palaeozoic archaeostracan carapaces |
| **Short Title:** | Archaeostracan hydrodynamics |
| **Corresponding Author:** | Stephen Pates<br>University of Cambridge<br>Cambridge, Cambridgeshire UNITED KINGDOM |
| **Keywords:** | Caryocaridid; archaeostracan; Ordovician; zoonekton; pelagic |
| **Abstract:** | The diversification of macroscopic pelagic arthropods such as caryocaridid archaeostracans was a crucial aspect of the Great Ordovician Biodiversification Event, and the plankton revolution. A pelagic mode of life has been inferred for caryocaridids from their common presence in black graptolitic shales alongside carapace morphologies that appear streamlined. However the hydrodynamic performance within the group and comparisons with other archaeostracans were lacking. Here we use a computational fluid dynamics approach to quantify the hydrodynamic performance of caryocaridids, and other early Palaeozoic archaeostracans including Arenosicaris inflata and Ordovician ceratiocaridids. We show that streamlining of the carapace was an important factor facilitating a pelagic mode of life in caryocaridids. However, comparable hydrodynamic performance is also recovered for some nektobenthic ceratiocaridids. This suggests that alongside carapace streamlining, adaptations to appendages and thinning of the carapace were also important for a pelagic mode of life in Ordovician caryocaridids. |
| **Order of Authors:** | Stephen Pates |
| | Yuan Xue |
| **Additional Information:** | |
| **Question** | **Response** |
| **Financial Disclosure**<br><br>Enter a financial disclosure statement that describes the sources of funding for the work included in this submission. Review the submission guidelines for detailed requirements. View published research articles from *PLOS ONE* for specific examples.<br><br>This statement is required for submission and **will appear in the published article** if the submission is accepted. Please make sure it is accurate. | SP received funding through a Herchel Smith Postdoctoral Fellowship (no number).<br>https://www.herchelsmith.cam.ac.uk/postdoctoral-fellowships<br>The sponsors had no role in study design, data collection and analysis, decision to publish, or preparation of the manuscript. |

Powered by Editorial Manager® and ProduXion Manager® from Aries Systems Corporation

| | |
|---|---|
| **Unfunded studies**<br>Enter: *The author(s) received no specific funding for this work.*<br><br>**Funded studies**<br>Enter a statement with the following details:<br>• Initials of the authors who received each award<br>• Grant numbers awarded to each author<br>• The full name of each funder<br>• URL of each funder website<br>• Did the sponsors or funders play any role in the study design, data collection and analysis, decision to publish, or preparation of the manuscript?<br>• **NO** - Include this sentence at the end of your statement: *The funders had no role in study design, data collection and analysis, decision to publish, or preparation of the manuscript.*<br>• **YES** - Specify the role(s) played.<br><br>* typeset | |
| **Competing Interests**<br><br>Use the instructions below to enter a competing interest statement for this submission. On behalf of all authors, disclose any competing interests that could be perceived to bias this work—acknowledging all financial support and any other relevant financial or non-financial competing interests.<br><br>This statement is required for submission and **will appear in the published article** if the submission is accepted. Please make sure it is accurate and that any funding sources listed in your Funding Information later in the submission form are also declared in your Financial Disclosure statement.<br><br>View published research articles from *PLOS ONE* for specific examples. | The authors have declared that no competing interests exist. |

*Powered by Editorial Manager® and ProduXion Manager® from Aries Systems Corporation*

| | |
|---|---|
| **NO authors have competing interests**<br><br>Enter: *The authors have declared that no competing interests exist.*<br><br>**Authors with competing interests**<br><br>Enter competing interest details beginning with this statement:<br><br>*I have read the journal's policy and the authors of this manuscript have the following competing interests: [insert competing interests here]*<br><br>* typeset | |
| **Ethics Statement**<br><br>Enter an ethics statement for this submission. This statement is required if the study involved:<br><br>• Human participants<br>• Human specimens or tissue<br>• Vertebrate animals or cephalopods<br>• Vertebrate embryos or tissues<br>• Field research<br><br>Write "N/A" if the submission does not require an ethics statement.<br><br>General guidance is provided below. Consult the submission guidelines for detailed instructions. **Make sure that all information entered here is included in the Methods section of the manuscript.** | NA |

| | |
|---|---|
| **Format for specific study types**<br><br>**Human Subject Research (involving human participants and/or tissue)**<br>• Give the name of the institutional review board or ethics committee that approved the study<br>• Include the approval number and/or a statement indicating approval of this research<br>• Indicate the form of consent obtained (written/oral) or the reason that consent was not obtained (e.g. the data were analyzed anonymously)<br><br>**Animal Research (involving vertebrate animals, embryos or tissues)**<br>• Provide the name of the Institutional Animal Care and Use Committee (IACUC) or other relevant ethics board that reviewed the study protocol, and indicate whether they approved this research or granted a formal waiver of ethical approval<br>• Include an approval number if one was obtained<br>• If the study involved *non-human primates*, add *additional details* about animal welfare and steps taken to ameliorate suffering<br>• If anesthesia, euthanasia, or any kind of animal sacrifice is part of the study, include briefly which substances and/or methods were applied<br><br>**Field Research**<br><br>Include the following details if this study involves the collection of plant, animal, or other materials from a natural setting:<br>• Field permit number<br>• Name of the institution or relevant body that granted permission | |
| **Data Availability**<br><br>Authors are required to make all data underlying the findings described fully available, without restriction, and from the time of publication. PLOS allows rare exceptions to address legal and ethical concerns. See the PLOS Data Policy and FAQ for detailed information. | Yes - all data are fully available without restriction |

Powered by Editorial Manager® and ProduXion Manager® from Aries Systems Corporation

| | |
|---|---|
| A Data Availability Statement describing where the data can be found is required at submission. Your answers to this question constitute the Data Availability Statement and **will be published in the article**, if accepted.<br><br>**Important:** Stating 'data available on request from the author' is not sufficient. If your data are only available upon request, select 'No' for the first question and explain your exceptional situation in the text box.<br><br>Do the authors confirm that all data underlying the findings described in their manuscript are fully available without restriction? | |
| **Describe where the data may be found in full sentences. If you are copying our sample text, replace any instances of XXX with the appropriate details.**<br><br>• If the data are **held or will be held in a public repository**, include URLs, accession numbers or DOIs. If this information will only be available after acceptance, indicate this by ticking the box below. For example: *All XXX files are available from the XXX database (accession number(s) XXX, XXX.*).<br>• If the data are all contained **within the manuscript and/or Supporting Information files**, enter the following: *All relevant data are within the manuscript and its Supporting Information files.*<br>• If neither of these applies but you are able to provide **details of access elsewhere**, with or without limitations, please do so. For example:<br><br>*Data cannot be shared publicly because of [XXX]. Data are available from the XXX Institutional Data Access / Ethics Committee (contact via XXX) for researchers who meet the criteria for access to confidential data.*<br><br>*The data underlying the results presented in the study are available from (include the name of the third party* | Data have been uploaded to an OSF project. Link:<br>https://osf.io/jv32t/?view_only=12216291e72e4b8393f3d46eb34fcc69 |

| | |
|---|---|
| *and contact information or URL).*<br>• This text is appropriate if the data are owned by a third party and authors do not have permission to share the data.<br><br>* typeset | |
| Additional data availability information: | Tick here if the URLs/accession numbers/DOIs will be available only after acceptance of the manuscript for publication so that we can ensure their inclusion before publication. |

Powered by Editorial Manager® and ProduXion Manager® from Aries Systems Corporation

Main text and figures Click here to access/download;Manuscript;PatesXue.docx

# Hydrodynamic performance of early Palaeozoic archaeostracan carapaces

Stephen Pates [1,2] * and Yuan Xue [2]

1. Department of Zoology, University of Cambridge, Downing Street, Cambridge, CB2 3EJ
2. Homerton College, University of Cambridge, Hills Road, CB2 8PH

*author for correspondence, sp587@cam.ac.uk

## Abstract

The diversification of macroscopic pelagic arthropods such as caryocaridid archaeostracans was a crucial aspect of the Great Ordovician Biodiversification Event, and the plankton revolution. A pelagic mode of life has been inferred for caryocaridids from their common presence in black graptolitic shales alongside carapace morphologies that appear streamlined. However the hydrodynamic performance within the group and comparisons with other archaeostracans were lacking. Here we use a computational fluid dynamics approach to quantify the hydrodynamic performance of caryocaridids, and other early Palaeozoic archaeostracans including *Arenosicaris inflata* and Ordovician ceratiocaridids. We show that streamlining of the carapace was an important factor facilitating a pelagic mode of life in caryocaridids. However, comparable hydrodynamic performance is also recovered for some nektobenthic ceratiocaridids. This suggests that alongside carapace streamlining, adaptations to appendages and thinning of the carapace were also important for a pelagic mode of life in Ordovician caryocaridids.

## Key words

Caryocaridid, archaeostracan, Ordovician, zoonekton, pelagic

## Introduction

Arthropods are a major component of the pelagic realm, functioning as intermediaries between primary producers and tertiary consumers in marine food webs (1). [illegible]oing so, they play key roles as ecosystem engineers, repackaging nutrients and organic matter as faecal pellets that are transported vertically through the biological pump (2–4) and biologically mixing the water column (5,6). Indeed the early Palaeozoic expansion and diversification of arthropods into the water column was one of a series of evolutionary and environmental feedbacks that transformed the oceans from a stratified, micro-organism dominated, water column with oxygen available only near the surface, to a well ventilated one where an animal-dominated biological pump links benthic and pelagic realms (1,6–12).

While pelagic arthropods are known from the early Cambrian (1,7,8,11) the synchronous radiation of phytoplankton, zooplankton and zoonekton with benthic suspension feeders as part of the plankton revolution in the late Cambrian to early Ordovician led to a further ecological revolution in early Palaeozoic oceans (13,14). Arthropods were critical at a number of different size and trophic classes within this ecological revolution, from microscopic planktonic trilobite larvae (15) to large meter scale filter feeders (16,17).

Ordovician arthropod macrozooplankton and macrozoonekton (size range 20-200 mm) (1) include trilobites (18,19) and caryocaridid archaeostracans (20,21). The morphological characteristics and cosmopolitan distribution of caryocaridids, including their carapace design and common presence in outer shelf or slope settings alongside graptolites in black shales, supports a pelagic life mode at the fringes of continental margins for this group (20–23).

In this study we explore the hydrodynamic performance (drag and lift) of caryocaridid carapaces, contrasting them with archaeostracan relatives including the late Cambrian benthic *Arenosicaris* (24) and Ordovician ceratiocaridids, which are generally considered nektobenthic. Thus we determine the role of the carapace in facilitating a pelagic mode of life in Ordovician caryocaridids.

## Materials and Methods

*Carapace outlines*

Two dimensional outlines of 16 species of Ordovician and late Cambrian archaeostracans were generated from reconstructions in the literature ((25), using Inkscape 1.0. These were then scaled, centered and aligned, before being sampled to the same resolution (64 points) and converted to a text file that could be read directly into ANSYS DesignModeler (Ansys Academic 2021 Release R2), using R (26), Momocs package (27) and a function provided by (11). Some shapes had rows of small posterior spines which created spaces too small for the meshing program to work, while for others sampling the outline at 64 points led to the anterior spine not being captured faithfully. For these shapes, outlines were modified to remove small posterior spines (but leaving in the dorsalmost and ventralmost spines) and sampled at 256 points in order to capture anterior spine morphology. All R code and outlines are provided in the accompanying OSF project (25).

*Computational Fluid Dynamics*

Following import into ANSYS DesignModeler, models were scaled to a length of 25mm. The geometry, meshing and model set-up of (11) were used as this has been validated and verified, and assessed the hydrodynamic performance of carapace valves of similar morphology and size (**Figure 2**). A dorsal length of 25mm was used to facilitate size-independent comparisons of hydrodynamic performance, and facilitate comparisons with this previous study. CFD simulations were conducted using the steady-state laminar solver in ANSYS Fluent (Ansys Academic, Release 2021 R2).

Forces and coefficients of drag and lift were calculated for a flow speed of 1 carapace length (25 mm) per second. The carapace length (25 mm) was taken as the reference area for calculation of coefficients. Solutions were considered converged when residuals were less than $10^{-6}$. Simulations were conducted at a range of angles of attack at 2 degree increments, to find the stall angle and

minimum drag generated by each carapace outline. If a solution did not converge (residuals remained greater than $10^{-6}$ and sinusoidal drag and lift forces generated across iterations) the flow was considered to be unsteady, and no drag or lift values were recorded. A further simulation at 1 degree less angle of attack was then conducted, to find the stall angle as accurately as possible. Simluations were performed at negative angles of attack until flow was unsteady or the drag coefficient was equal to or greater than that at the stall angle.

*Coefficients and calculations*

Coefficients of drag and lift ($C_d$, $C_l$) were calculated for each converged simulation. Coefficients of drag and lift are proportional to the force (either drag or lift, $F_d$ and $F_l$ respectively), inverse square of swimming speed (u) and the inverse of the reference length (A) (**Equation 1 and 2;** ρ is the density of the fluid).

$$C_d = \frac{2F_d}{\rho u A} \text{ [Equation 1]}$$

$$C_l = \frac{2F_l}{\rho u A} \text{ [Equation 2]}$$

Drag:lift ratios were calculated by dividing the drag coefficient by the lift coefficient at the same angle of attack.

## Results

*Mesh sensitivity*

In mesh sensitivity analyses, results were considered mesh independent if the values were less than 2% from the more refined mesh. A refinement 1 mesh (35-40,000 elements) was chosen for all carapaces, as in all cases these results were within the mesh independent threshold compared to the refinement 2 mesh (c. 80-85,000 elements). In some cases results from the starting mesh wereless than 2% different to the refinement 1 mesh, however a refinement 1 mesh was still used in

these calculations as it was not significantly more computationally expensive, and meant that approximately the same number of elements were used for all taxa.

*Drag and lift coefficients*

Over 250 simulations were run. The highest number of simulations were run for *Jellicaris stewarti* (21 simulations), covering a range of 39 degrees angle of attack. The fewest were run for *Rolfecaris lethiersi* (6 simulations), over a range of 6 degrees angle of attack, of which three simulations converged, covering a range of 3 degrees angle of attack.

Archaeostracan carapaces display a broad range of drag and lift coefficients (**Figure 3**). The carapaces with the highest drag coefficients belonged to *Arenosicaris inflata* and two ceratiocaridids (*Pumilocaris granulosa* and *Rolfecaris lethiersi*). Drag coefficients were lower in all caryocaridids and the two *Ceratiocaris* species (*C. angusta* and *C. silicula*). All caryocaridids displayed broad ranges of lift coefficients including both negative and positive values, with the exception of *Janviericaris formosa* (positive values only). Lift coefficient ranges were broadest the two *Ceratiocaris* species, *Caryocaris acoitensis* and *Caryocaris zhejiangensis,* and *Saltericaris subula*.

*Pressure and velocity contours*

Carapace shape affected flow, which can be visualised by comparing the pressure and velocity contours around *Arenosicaris inflata* with a caryocaridid and ceratiocaridid (**Figures 4, 5**). The larger area of high pressure at the anterior of the *Arenosicaris inflata* carapace, compared to most hydrodynamic caryocaridids and ceratiocaridids (**Figure 4**), is likely responsible for the higher drag. Visualisation of the velocity contours for the same carapaces reveals a broader, more unsteady, and more elongate wake for *A. inflata* (**Figure 5**).

*Phylogenetic context*

Plots of minimum drag coefficient against highest lift:drag ratio demonstrate a likely relationship between these two metrics of hydrodynamic performance (**Figure 6**). Carapaces of taxa a higher

number of nodes away from *Arenosicaris inflata* (**Figure 1**) are broadly more hydrodynamic (higher lift:drag ratio, lower minimum drag coefficient) than those fewer nodes away. The sample size is too small to statistically test for correlation.

## Discussion

The carapaces of caryocaridids outperformed *Arenosicaris inflata* and two of four ceratiocaridids considered in terms of minimum drag coefficient, range of lift coefficients and lift:drag ratios. This indicates that improved hydrodynamic performance of the carapace was an important factor in facilitating a pelagic mode of life in Ordovician caryocaridids, providing quantitative support for qualitative assessments by previous workers (20,21). Lower drag coefficients were achieved through increasing the length relative to the depth of carapaces. The role of the anterior spine is less clear, as low drag coefficients were achieved by carapaces both with (e.g. *Ca. acoitensis, Ca. zhejiangensis*) and without (*Ce. Angusta, Ce. silicula*) anterior spines (**Figure 3**). Taxa with the highest drag coefficients lacked anterior spines and had broader carapaces relative to other archaeostracans (*Arenosicaris inflata, Pumilocaris granulosa, Rolfecaris lethiersi*; **Figure 3**).

*Janviericaris formosa* is notable amongst the caryocaridids for its relatively limited range of lift coefficients, which are all at positive values (**Figure 3**). These results indicate that the carapace was not well adapted for a pelagic mode of life. This is corroborated by the geological evidence, where *J. formosa* is found only in shelf deposits (28), contrasting to other caryocaridids which are found in slope and/or basin deposits (28). *Soomicaris cedabergensis*, interpreted as an epipelagic representative of the group (29), displays comparable hydrodynamic properties to mesopelagic *Caryocaris* taxa (**Figure 3**).

For archaeostracans, drag reduction would have facilitated more efficient (and possibly faster) swimming, which is important for both pelagic and nektobenthic life modes, as assuming these

arthropods were negatively buoyant, generation of lift would have also counteracted the negative buoyancy of the animals while swimming, again improving efficiency. This provides a possible explanation for the tentative link between phylogenetic distance from the benthic and burrowing *Arenosicaris* (24) and decreased drag and improved lift (**Figure 6**).

In this study, we focused on the role of the carapace, however this is just one of a number of morphological features that can improve swimming efficiency and speed in archaeostracans, or indeed any arthropod with an abdomen and specialised swimming appendages. Notably, the maximum lift, ranges of lift coefficients, and minimum drag coefficient for two Cambrian *Isoxys* carapaces (*I. longissimus* and *I. paradoxus ;* (11)) are more hydrodynamically favourable than the best archaeostracans in this study. This suggests that for a stem-group arthropod where detailed specialisation of appendages and development of an abdomen were not possible, adaptation of the carapace may have played a more important role.

Within archaeostracans, the abdomen may have played an important role in a number of ways. There is no evidence that it could dorsally flex (and so a 'power stroke' as in *Nebalia* may not have been a major way of providing thrust (22)), however it may have still been important for escape reactions (22). In addition, by elongating the body, the point of separation and swimming speed at which separation of the flow occurred would have been delayed. A functional comparison can be made to the elongate posterior spines are known in a likely pelagic Cambrian stem-group arthropod *Isoxys longissimus* (7,11,30,31). In *Isoxys* the body is nearly entirely covered by the carapace, and an abdomen not present (32) making elongate posterior spines a way of delaying the point of separation and thus improving hydrodynamic performance.

The appendage morphology was also likely important – indeed given the similar hydrodynamic performance of *Ceratiocaris* species in this study to pelagic caryocaridids, the additional thrust provided by specialised swimming appendages were likely important for facilitating a pelagic mode of life in the group (20,22).

## Conclusions

The carapace of pelagic caryocaridids generated broader ranges of lift coefficients and lower drag coefficients than the benthic *Arenosicaris inflata* at the same Reynolds number. However, some nektobenthic ceratiocaridids, such as *Ceratiocaris,* display comparable hydrodynamic performance. Thus modification of the carapace shape was likely an important factor facilitating a pelagic mode of life in caryocaridids, as well as a nektobenthic lifestyle for some ceratiocaridids. However, the overlap in hydrodynamic performance with some pelagic and nektobenthic taxa indicates that improvement in swimming power through adaptations to appendages, as well as thinning of the carapace, were also important for the explosion of caryocaridid diveristy into the water column during the Ordovician.

## Acknowledgements

SP acknowledges funding through a Herchel Smith Postdoctoral Fellowship (University of Cambridge).

## Author contributions

SP: conceptualization, methodology, validation, formal analysis, investigation, resources, data curation, writing – original draft, visualisation, supervision, project administration and funding acquisition.

YX: formal analysis, investigation, methodology, data curation, validation, writing – review & editing.

## Declaration of interests

The authors declare no competing interests.

## References


1. Perrier V, Williams M, Siveter DJ. The fossil record and palaeoenvironmental significance of marine arthropod zooplankton. Earth Sci Rev [Internet]. 2015;146:146–62. Available from: http://dx.doi.org/10.1016/j.earscirev.2015.02.003
2. Jones CG, Lawton JH, Shachak M. Organisms as ecosystem engineers. OIKOS. 1994;69:373–86.
3. Butterfield NJ. Animals and the invention of the Phanerozoic Earth system. Trends Ecol Evol. 2011 Feb 1;26(2):81–7.
4. Turner JT. Zooplankton fecal pellets, marine snow, phytodetritus and the ocean's biological pump. Prog Oceanogr [Internet]. 2015;130:205–48. Available from: http://dx.doi.org/10.1016/j.pocean.2014.08.005
5. Breitburg DL, Crump BC, Dabiri JO, Gallegos CL. Ecosystem Engineers in the Pelagic Realm: Alteration of Habitat by Species Ranging from Microbes to Jellyfish. Integr Comp Biol [Internet]. 2010 Aug 1 [cited 2023 Jul 17];50(2):188–200. Available from: https://dx.doi.org/10.1093/icb/icq051
6. Butterfield NJ. Oxygen, animals and aquatic bioturbation: An updated account. Geobiology. 2018;16(1):3–16.
7. Vannier J, Chen J yuan. The Early Cambrian colonization of pelagic niches exemplified by Isoxys (Arthropoda). Lethaia. 2000;33:295–311.
8. Vannier J, García-Bellido DC, Hu SX, Chen AL. Arthropod visual predators in the early pelagic ecosystem: Evidence from the Burgess Shale and Chengjiang biotas. Proceedings of the Royal Society B: Biological Sciences. 2009;276(1667):2567–74.
9. Lenton TM, Boyle RA, Poulton SW, Shields-Zhou GA, Butterfield NJ. Co-evolution of eukaryotes and ocean oxygenation in the Neoproterozoic era. Nat Geosci. 2014;7(4):257–65.
10. Williams M, Vandenbroucke TRA, Perrier V, Siveter DJ, Servais T. A link in the chain of the Cambrian zooplankton: Bradoriid arthropods invade the water column. Geol Mag [Internet]. 2015 Sep 6 [cited 2021 May 5];152(5):923–34. Available from: https://doi.org/10.1017/S0016756815000059
11. Pates S, Daley AC, Legg DA, Rahman IA. Vertically migrating *Isoxys* and the early Cambrian biological pump. Proceedings of the Royal Society B [Internet]. 2021 Jun 30 [cited 2022 Aug 15];288(1953). Available from: https://royalsocietypublishing.org/doi/10.1098/rspb.2021.0464
12. Meyer KM, Ridgwell A, Payne JL. The influence of the biological pump on ocean chemistry: implications for long-term trends in marine redox chemistry, the global carbon cycle, and marine animal ecosystems. Geobiology [Internet]. 2016 May 1 [cited 2023 Aug 14];14(3):207–19. Available from: https://onlinelibrary.wiley.com/doi/full/10.1111/gbi.12176

13. Servais T, Owen AW, Harper DAT, Kröger B, Munnecke A. The Great Ordovician Biodiversification Event (GOBE): The palaeoecological dimension. Vol. 294, Palaeogeography, Palaeoclimatology, Palaeoecology. Elsevier; 2010. p. 99–119.

14. Servais T, Perrier V, Danelian T, Klug C, Martin R, Munnecke A, et al. The onset of the 'Ordovician Plankton Revolution' in the late Cambrian. Palaeogeogr Palaeoclimatol Palaeoecol. 2016 Sep 15;458:12–28.

15. Laibl L, Saleh F, Pérez-Peris F. Drifting with trilobites: The invasion of early post-embryonic trilobite stages to the pelagic realm. Palaeogeogr Palaeoclimatol Palaeoecol. 2023 Mar 1;613:111403.

16. Van Roy P, Daley AC, Briggs DEG. Anomalocaridid trunk limb homology revealed by a giant filter-feeder with paired flaps. Nature [Internet]. 2015;522(7554):77–80. Available from: http://dx.doi.org/10.1038/nature14256

17. Potin GJM, Gueriau P, Daley AC. Radiodont frontal appendages from the Fezouata Biota (Morocco) reveal high diversity and ecological adaptations to suspension-feeding during the Early Ordovician. Front Ecol Evol [Internet]. 2023 Aug 9 [cited 2023 Aug 15];11:1214109. Available from: https://www.frontiersin.org/articles/10.3389/fevo.2023.1214109/full

18. Esteve J, López-Pachón M. Swimming and feeding in the Ordovician trilobite Microparia speciosa shed light on the early history of nektonic life habits. Palaeogeogr Palaeoclimatol Palaeoecol. 2023 Sep 1;625:111691.

19. Fortey RA. Pelagic trilobites as an example of deducing the life habits of extinct arthropods. Earth Environ Sci Trans R Soc Edinb [Internet]. 1985 [cited 2021 Aug 31];76(2–3):219–30. Available from: https://www.cambridge.org/core/journals/earth-and-environmental-science-transactions-of-royal-society-of-edinburgh/article/pelagic-trilobites-as-an-example-of-deducing-the-life-habits-of-extinct-arthropods/64C993D1AC8891D1FC5F4D28A7170C4E

20. Vannier J, Racheboeuf PR, Brussa ED, Williams M, Rushton AWA, Servais T, et al. Cosmopolitan arthropod zooplankton in the Ordovician seas. Palaeogeogr Palaeoclimatol Palaeoecol. 2003 Jun 5;195(1–2):173–91.

21. Liu Y, Fan R, Zong R, Gong Y. The evolution and initial rise of pelagic caryocaridids in the Ordovician. Earth Sci Rev. 2022 Aug 1;231:104097.

22. Vannier J, Boissy P, Racheboeuf PR. Locomotion in Nebalia bipes: a possible model for Palaeozoic phyllocarid crustaceans. Lethaia [Internet]. 1997 Jun 1 [cited 2023 Aug 15];30(2):89–104. Available from: https://onlinelibrary.wiley.com/doi/full/10.1111/j.1502-3931.1997.tb00449.x

23. Chlupac I. Phyllocarid crustaceans from the Middle Ordovician Šárka Formation at Praha-Vokovice. Bull Geosci. 2003;78(2):107–11.

24. Collette JH, Hagadorn JW. Three-dimensionally preserved arthropods from Cambrian Lagerstatten of Quebec and Wisconsin. J Paleontol [Internet]. 2010 Jul 1 [cited 2022 Oct 6];84(4):646–67. Available from: http://pubs.geoscienceworld.org/jpaleontol/article-pdf/84/4/646/3035284/i0022-3360-84-4-646.pdf

25. Pates S, Xue Y. Open Science Framework. 2023 [cited 2023 Aug 17]. OSF project. Available from: https://osf.io/jv32t/?view_only=12216291e72e4b8393f3d46eb34fcc69

26. R Core Team. R: A language and environment for statistical computing. R Foundation for Statistical Computing, Vienna, Austria. 2020. R Core Team. Available from: http://www.r-project.org/

27. Bonhomme V, Picq S, Gaucherel C, Claude J. Momocs: Outline analysis using R. J Stat Softw. 2014;56(13):1–24.

28. Liu Y long, Fan R ying, Zong R wen, Gong Y ming. First occurrence of caryocaridids (Crustacea, Phyllocarida) in the Ordovician of North China. Palaeogeogr Palaeoclimatol Palaeoecol. 2023 Aug 1;623:111638.

29. Whittle RJ, Gabbott SE, Aldridge RJ, Theron JN. Taphonomy and palaeoecology of a Late Ordovician caryocaridid from the Soom Shale Lagerstätte, South Africa. Palaeogeogr Palaeoclimatol Palaeoecol. 2007 Aug 8;251(3–4):383–97.

30. García-Bellido DC, Vannier J, Collins D. Soft-part preservation in two species of the arthropod *Isoxys* from the middle Cambrian Burgess Shale of British Columbia, Canada. Acta Palaeontol Pol. 2009;54(4):699–712.

31. Lerosey-Aubril R, Kimmig J, Pates S, Skabelund J, Weug A, Ortega-Hernández J. New exceptionally preserved panarthropods from the Drumian Wheeler Konservat-Lagerstätte of the House Range of Utah. Pap Palaeontol. 2020;6(4):501–31.

32. Zhang C, Liu Y, Ortega-Hernández J, Wolfe JM, Jin C, Mai H, et al. Three-dimensional morphology of the biramous appendages in *Isoxys* from the early Cambrian of South China, and its implications for early euarthropod evolution. Proceedings of the Royal Society B [Internet]. 2023 Apr 26 [cited 2023 Jun 1];290(1997):20230335. Available from: https://royalsocietypublishing.org/doi/10.1098/rspb.2023.0335

## Figures and captions

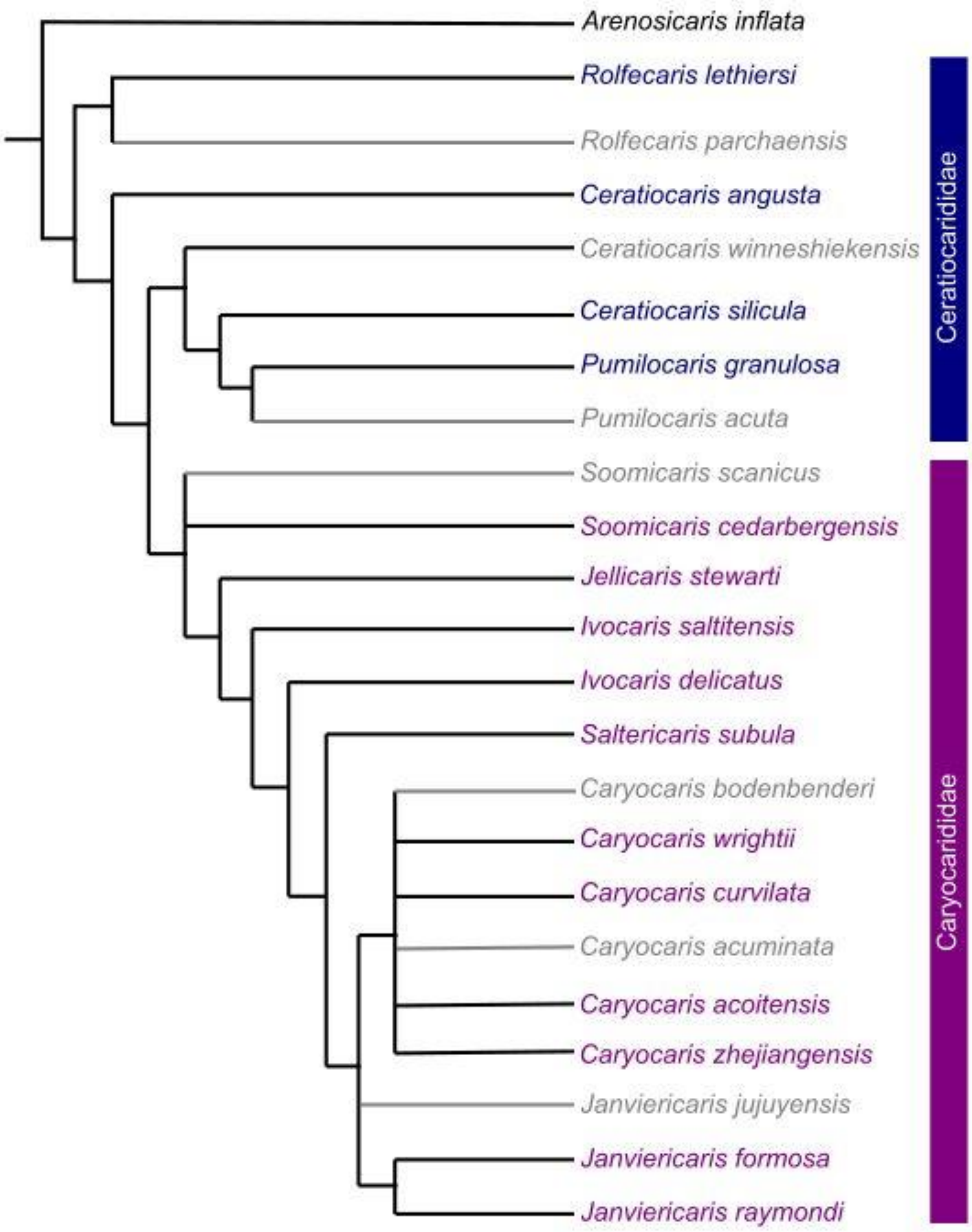


**Figure 1.** Cladogram of Ordovician archaeostracans (adapted from Liu et al. 2022, fig. 2). Taxa not selected for computational fluid dynamics analyses in grey.

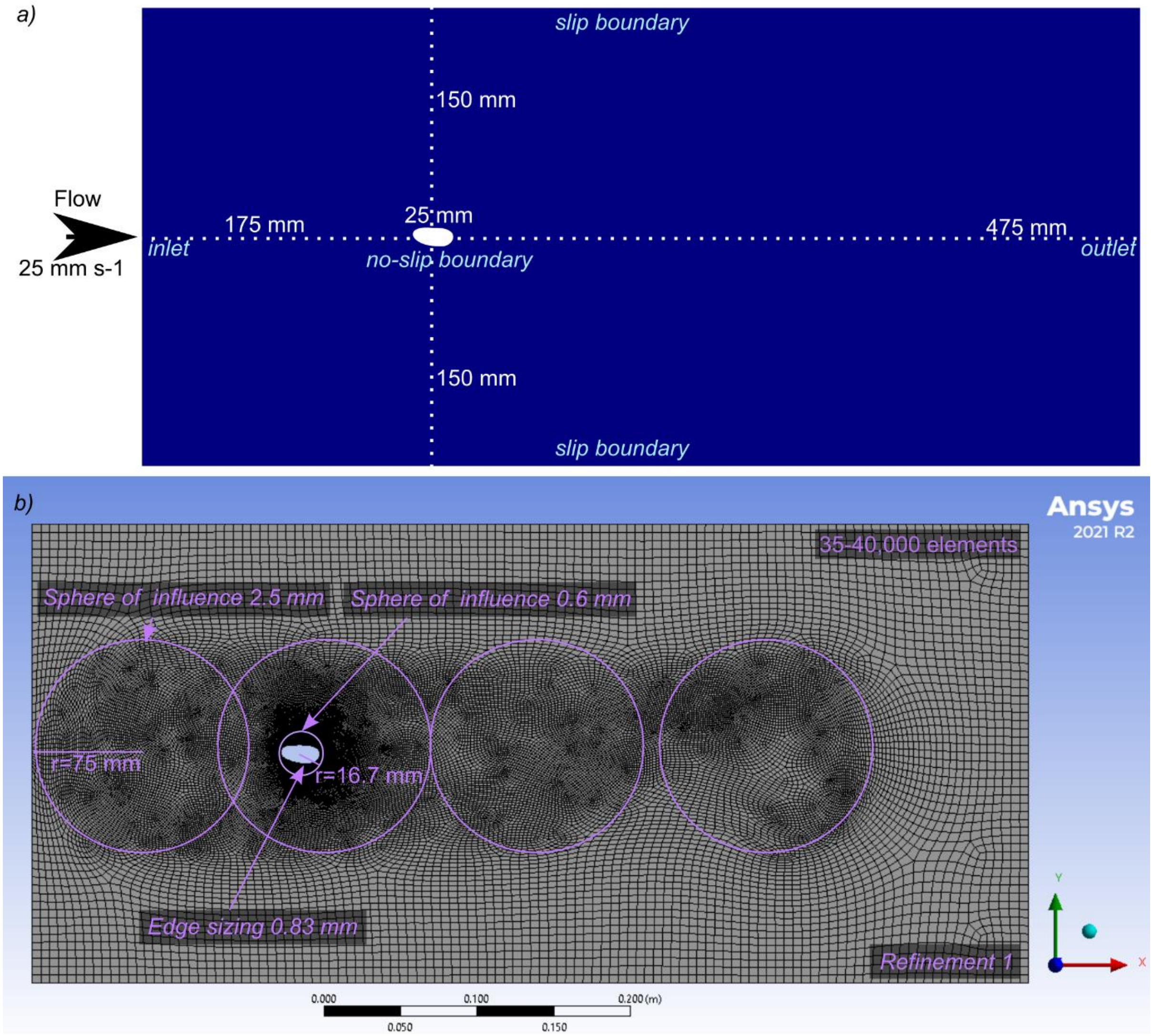


**Figure 2.** Geometry and boundary conditions for simulations (a) and meshing set up (b) used in this study. Element sizes given next to meshing controls. Whole domain subjected to Refinement 1 (Ansys Meshing; Ansys Academic 2021 release R2).

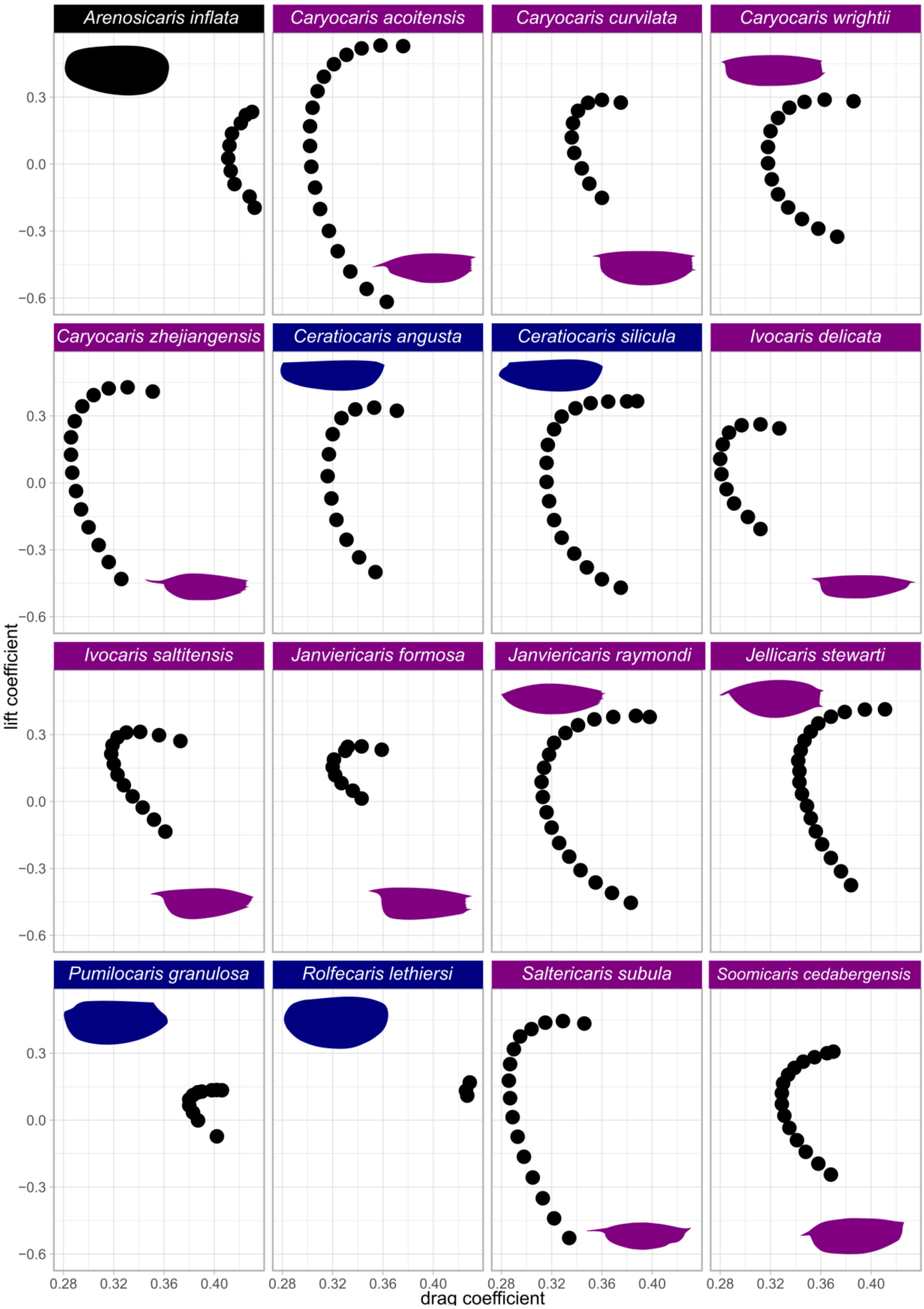


**Figure 3**. Drag polars for all taxa in this study, overlain by carapace outline used in computational fluid dynamics simulation. Each point represents results (drag and lift coefficients) of a single

simulation at Reynolds number of 340. Full results including mesh refinement provided in OSF project, alongside ANSYS project files(25).

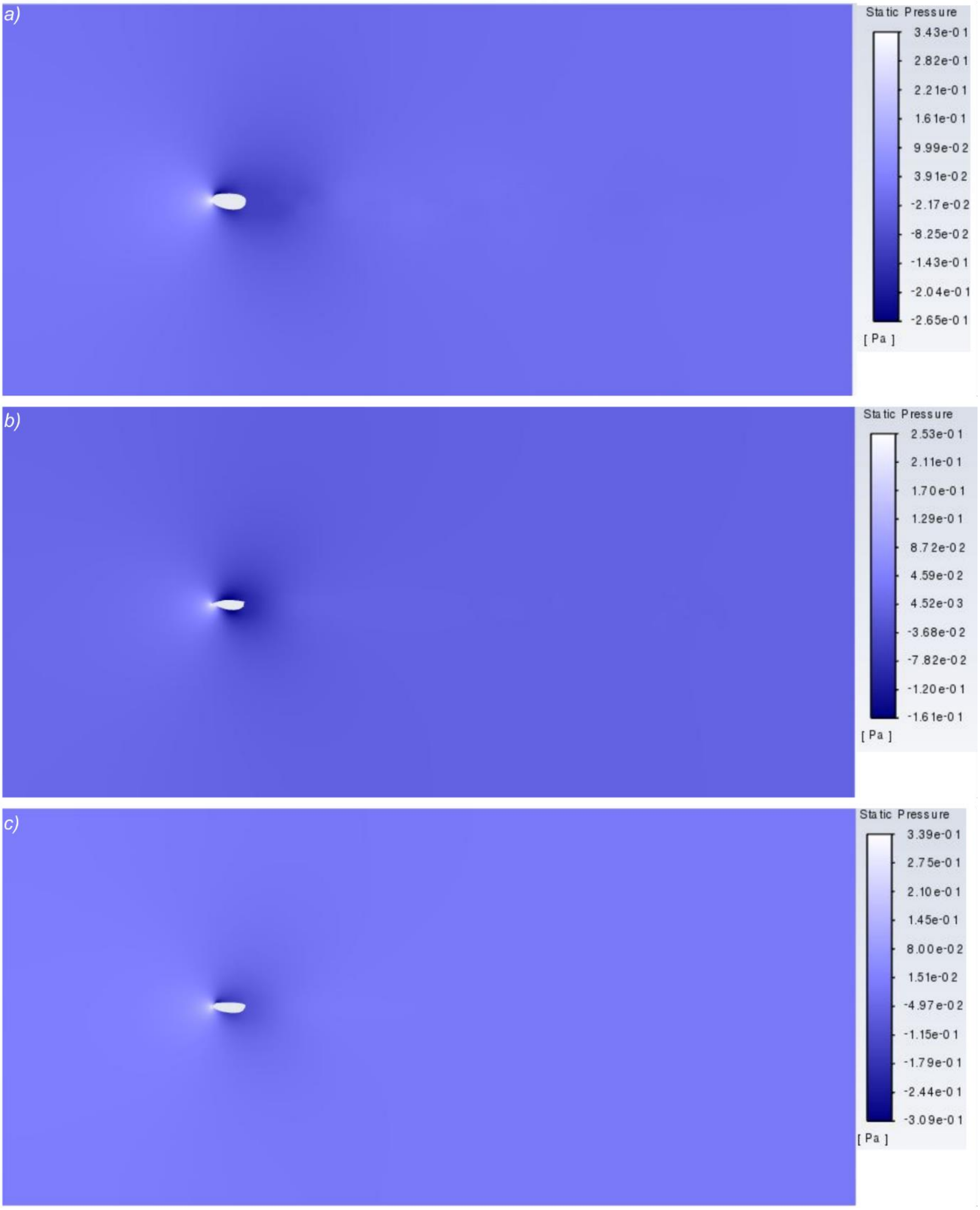


**Figure 4.** Contours of static pressure for carapaces of *Arenosicaris inflata* (a) *Caryocaris acoitensis* (b) and *Ceratiocaris silicula* (c). Angle of attack for all 0 degrees. Visualized using ANSYS Fluent. Length of carapaces 25 mm.

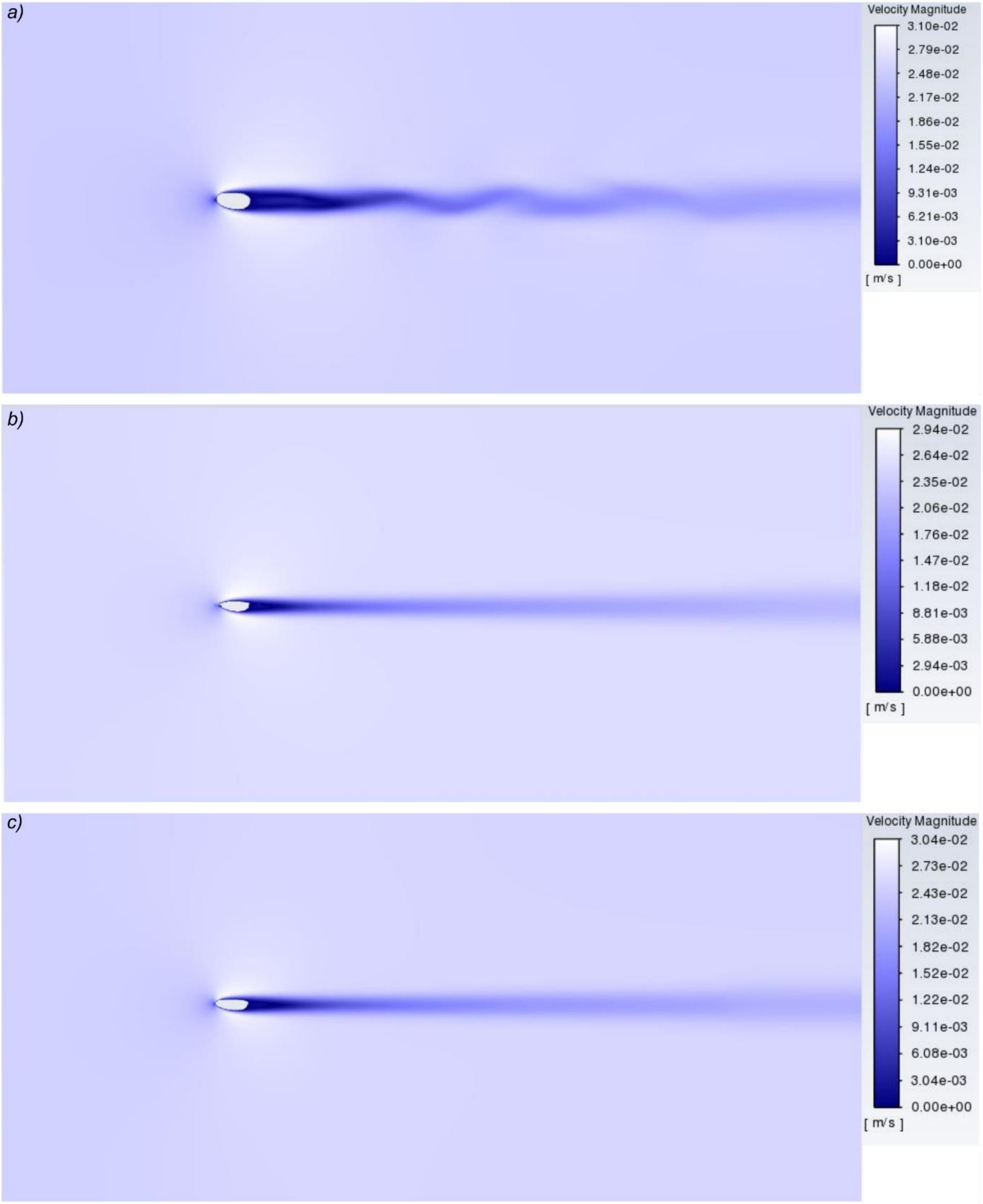


**Figure 5**. Contours of velocity magnitude or carapaces of *Arenosicaris inflata* (a) *Caryocaris acoitensis* (b) and *Ceratiocaris silicula* (c). Angle of attack for all 0 degrees. Visualized using ANSYS Fluent. Length of carapaces 25 mm.

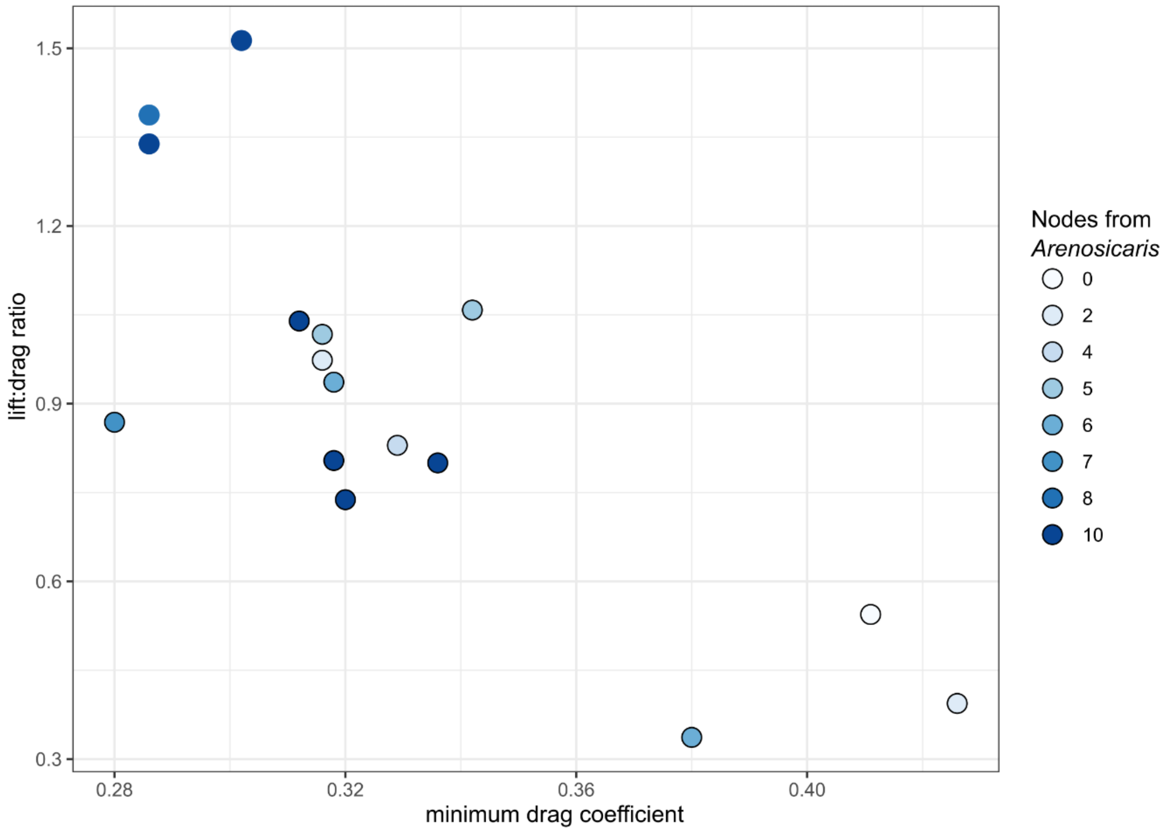


**Figure 6.** Plot of minimum drag coefficient against highest lift:drag ratio for all carapaces analysed in this study. Color of point indicates number of nodes away from *Arenosicaris inflata* (according to cladogram presented in **Figure 1,** which is adapted from Liu et al. 2022, figure 2).
