# Supplementary material for: Hydrodynamic performance of Ordovician archaeostracan carapaces
Source: PLoS One. 2024 May 31;19(5):e0304559. doi: 10.1371/journal.pone.0304559 (PMC11142683; doi:10.1371/journal.pone.0304559)
Supplement: Attachment — Submitted filename: Response_to_reviewers.docx [file pone.0304559.s006.docx]

**PONE-D-23-26410**

**Hydrodynamic performance of early Palaeozoic archaeostracan carapaces**

**PLOS ONE**

Dear Dr. Pates,

Thank you for submitting your manuscript to PLOS ONE. After careful consideration, we feel that it has merit but does not fully meet PLOS ONE&rsquo;s publication criteria as it currently stands. Therefore, we invite you to submit a revised version of the manuscript that addresses the points raised during the review process.

Please submit your revised manuscript by Apr 04 2024 11:59PM. If you will need more time than this to complete your revisions, please reply to this message or contact the journal office at [plosone@plos.org](mailto:plosone@plos.org). When you&#39;re ready to submit your revision, log on to https://www.editorialmanager.com/pone/ and select the &#39;Submissions Needing Revision&#39; folder to locate your manuscript file.

Please include the following items when submitting your revised manuscript:

- A rebuttal letter that responds to each point raised by the academic editor and reviewer(s). You should upload this letter as a separate file labeled &#39;Response to Reviewers&#39;.
- A marked-up copy of your manuscript that highlights changes made to the original version. You should upload this as a separate file labeled &#39;Revised Manuscript with Track Changes&#39;.
- An unmarked version of your revised paper without tracked changes. You should upload this as a separate file labeled &#39;Manuscript&#39;.

If you would like to make changes to your financial disclosure, please include your updated statement in your cover letter. Guidelines for resubmitting your figure files are available below the reviewer comments at the end of this letter.

If applicable, we recommend that you deposit your laboratory protocols in protocols.io to enhance the reproducibility of your results. Protocols.io assigns your protocol its own identifier (DOI) so that it can be cited independently in the future. For instructions see: <https://journals.plos.org/plosone/s/submission-guidelines#loc-laboratory-protocols>. Additionally, PLOS ONE offers an option for publishing peer-reviewed Lab Protocol articles, which describe protocols hosted on protocols.io. Read more information on sharing protocols at [https://plos.org/protocols?utm_medium=editorial-email&amp;utm_source=authorletters&amp;utm_campaign=protocols](https://plos.org/protocols?utm_medium=editorial-email&utm_source=authorletters&utm_campaign=protocols).

We look forward to receiving your revised manuscript.

Kind regards,

Shuan-Hong Zhang, PhD
Academic Editor
PLOS ONE

Dear Editor

We thank the editor and the two reviewers for their thoughtful and detailed comments on the manuscript. We have made changes to the text and run additional analyses in order to address them, and they have improved this work. The OSF project that provides all the code, underlying data, and results from the simulations (as well as the archived ANSYS project files) has now been made public, with doi.org/10.17605/OSF.IO/JV32T.

In our opinion this manuscript is now ready for publication, following the changes resulting from peer review.

Many thanks,

Stephen Pates and Yuan Xue

**Review Comments to the Author**

**Reviewer #1**: Dear authors,
I found your research very interesting and of broad interest. However, there is, in my opinion a significant problem that, at the very least, needs to be discussed: you used 2D outlines from reconstructions published in several articles from the literature. There are two main issues with this. First, you cannot know if the reconstructions are made using comparable methods, so that species may not be comparable. Second, and worse, some species you used are not fully known in their outline and those reconstructions extrapolate the missing outline, that is the case for J. raymondi and C. angusta. For J. raymondi, we lacks at least 1/4th of the carapace anteriorly.
I think it is necessary to correct these issues, clearly state in the manuscript how many simulations were made, and what are the results for each species.
Sincerely

We thank Reviewer #1 for their constructive comments, and detailed notes in the attached pdf.

We have added a section to the methods *Taxonomic sampling* where we reference the studies where idealised outlines were taken from. All but two outlines were from Liu et al. 2022 *Earth Science Reviews*. The reviewer is correct to point out that two outlines were made from incompletely known material. This is now highlighted in the section *Carapace outlines*.

A new table, Table 1, has been added to the manuscript with the number of simulations performed for each species. The results for each species are found in Figure 3, and also provided in .csv and .xlsx format on the OSF project that is cited in numerous places in the text. This project has now been made public, with a doi (doi.org/10.17605/OSF.IO/JV32T), and cited in the reference list as is considered good practice.

We have copied the comments from the pdf (black text) and indicated where we have made changes (red text) below:

What about connection between the planktonic realm and benthic realm? Arthropods also do that. Your second sentence seems to derive from the first, yet, in the first you explain they are a link in the food chain by eating photosynthetic organisms and being eaten by larger animals. In the second sentence, we don't find that concept, but rather other function of arthropods. Maybe this can be rephrased.

We have rephrased this start of the introduction, including mention of the link between benthic and pelagic realms in the first sentence.

Why just outlines? Don't you think a 3D model would have been much more accurate?

The use of 2D outlines allows us to focus on differences in morphology that can be observed from fossil data. It also allows us to include a wide range of taxa, some of which are known only from lateral compressions. Making 3D models of this number of taxa with confidence would not be possible, and would introduce additional variables and unknowns. In our opinion, in this study value can be gained from considering the 2D outlines, and focusing on the differences that can be observed in the shape. That is not to say that a 3D approach would not also be of value.

We have added a section to the Methods called *Benefits and limits of a 2D approach* where we highlight the benefits and limits of our 2D approach.

This is my biggest worry about this work. I think your method has value, but using reconstructions questions most of your results: how do you know how accurate the reconstructions are. They were probably not created to run physics models on. Also, For C. angusta and J. raymondi, I checked Liu 2022, and the reconstruction is incomplete, meaning you used a shape extrapolated from incomplete data... This is quite terrible for how confident you can be in your results. You should at the very least discuss this issue and explain why you think it is possible to use extrapolation in this context.

This is an important point to highlight. In our view, the reconstructions are suitable for considering differences in the 2D morphology between species, and the impact of this on the hydrodynamics of these animals. Our reasoning for this is now included in the subsection *Carapace outlines*. In addition, we make particular note of the incomplete taxa – *C. angusta* and *J. raymondi*. We agree that the results from these two taxa should be treated with more caution than the others in the study.

I have not used this technique before. I think I understand what you did, but it seems strange to convert shapes to a text. Perhaps you can give a little more explanations about what is happening here.

ANSYS can read in coordinates as a .txt file. We have added a short part of a sentence to make this clearer.

IS there a reason why you choose this particular size? Why not 30 mm, 10 mm? Does that correspond to the usual size of your studied organisms?

Another important point to consider. It is necessary to standardise the size of the shapes in the simulations in order to focus solely on the impact of shape, rather than have two variables (size and shape). We have added documentation of the size ranges of taxa in the study to subsection *Computational Fluid Dynamics* with references to the literature, to show that this size is close to or within the reported ranges of the taxa of interest. Note that the interpretation of the results would likely be very similar if a carapace length standard size of 30 mm was chosen, as the Reynolds number would be very similar.

You should cite fig 1. first.

Figure 1 is now cited in a new subsection *Taxonomic sampling* in the methods.

Any particular reason you selected this particular value?

This swimming speed was chosen as it falls within the range reported from extant marine crustaceans of a similar size. A supporting reference and explanation of this has been added to the text in Methods subsection *Computational Fluid Dynamics* . Reference to a previous study also shows that slight differences in the swimming speed will lead to similar relative results between taxa and thus similar interpretations.

Considering how important the meshing is, maybe you could have given a very short explanation rather that just

The explanation of the meshing procedure has been expanded (Methods subsection *Computational Fluid Dynamics*). Full details are visible in Figure 2, and also available through the ANSYS project archives in the OSF project.

You should include some data about this: comparisons between the two maybe?

The reasons behind this threshold of 2% have been expanded upon in this part of the Results.

We need details for each species.

A new table, Table 1, provides the number of simulations for each species.

We would also need to have the reference for the source of data for each carapace outline in the main text.

These are provided in the Methods *Taxonomic sampling* subsection.

I am not sure to understand. Maybe worth reformulating.

This sentence has been removed, with the full details now provided in Table 1.

It seems the references are not homogeneous. Some journal article have a mention of when they where consulted, which is not relevant, as these do not change, some have a mention "internet" for some reason. IT would be better to check carefully all this reference list.

This resulted from use of the Mendeley citation package, with the PloS format. We have manually corrected the references to remove these erroneous pieces of information.

**Reviewer #2**: Manuscript PONE-D-23-26410 reports on the hydrodynamic performance of Cambro-Ordovician archaeostracan carapaces to discuss their lifestyle and ecological evolution across the Great Ordovician Biodiversification Event. The paper is overall well-written and well-illustrated, and represents an interesting contribution on the topic.

There is, however, an important issue with the antero-posterior orientation of the carapaces that absolutely needs to be fixed before this work can be considered for publication: most carapaces are oriented with the anterior region on the left, but Ceratiocaris silicula and Ceratiocaris angusta are represented antero-posteriorly inverted (i.e. with the anterior region on the right). The error probably comes from the erroneous use of the drawings in fig. 4 of Liu et al. 2022 (your ref. 21) that shows left valves for A and B (Pumilocaris granulosa &amp; Pumilocaris salina) but right valves for Ceratiocaris silicula &amp; Ceratiocaris angusta. I feel that there is also a similar problem of orientation for Arenosicaris inflata following fig. 11.1 of Collette &amp; Hagadorn 2010 (your ref. 24).
Please re-run the analyses with the correct orientation. I guess, because the paper is short, that you could even use this &ldquo;mistake&rdquo; to further discuss the impact of such errors of orientation in CFD analyses, typically by comparing your current CFD results for the erroneous orientation (posterior to the left) with the results obtained for the correct orientation (anterior to the left).
We thank the reviewer for their careful review, and for spotting this error. We have rerun the analyses for *Arenosicaris inflata, Ceratiocaris angusta* and *Ce. silicula*, with the carapaces in the correct orientation. These results have led to updated figures 3 – 6, and have been updated in the accompanying OSF project.

I am also listing below additional minor suggestions/corrections (suggestions mostly towards giving a bit more explanations on the interpretation of the CFD data for paleontologists who are not familiar (yet!) with the approach):

- I would replace &ldquo;early Palaeozoic&rdquo; in the title with &ldquo;Ordovician&rdquo; for it be be more self-explainatory

We have made this change. Initially we used early Palaeozoic as *Arenosicaris* is Cambrian, however as the discussion focuses on Ordovician caryocaridids (in the most part) this more refined title is appropriate.

- Ref. 22 &ldquo;Nebalia bipes&rdquo; should be italicized

Changed.

- line 52, drop a bracket in &ldquo;((25)&rdquo;

Changed.

- line 54 drop the coma in &ldquo;using R (26), Momocs package (27)&rdquo;

Changed.

- line 61 You justify later (lines 64&ndash;66) the choice of scaling all models to the same length of 25mm, but considering that the size of caryocaridids was highly variable (and considering also that the paper is short) maybe also elaborate a little on the impact of size on the performed analyses (we can expect that some -if not most- of the readers might not be familiar with Computational Fluid Dynamics).

While size will impact the exact results, it will not impact the relative results as much. The chosen size has been justified further (following comments from Reviewer 1) and showing that for the taxa in this study it is appropriate.

- lines 63-64 refers to Figure 2 while Figure 1 has not been called yet. Figure 1 is actually not called at all in the text. Refer to Figure 1 in the previous paragraph (&ldquo;Carapace outlines&rdquo;) or, and it might be better, add a Methods paragraph about &ldquo;Taxonomic sampling&rdquo; that refers to Figure 1 and justify the choice / reject of the taxa.

We now have a methods subsection *Taxonomic sampling* as suggested, and refer to Figure 1 there. We justify the choice of taxa in this new subsection.

- line 88 paragraph &ldquo;Mesh sensitivity&rdquo;: because you have two different types of meshing presented in the Methods section, one for the generation of your 64-point carapace outline and the other for the CFD analyses, please remind here the one you are talking about
We have changed the title of this subsection to include the words CFD and mention computational fluid dynamics in the first sentence, in order to improve the clarity.

- line 93 &ldquo;wereless&rdquo; lacks a space.

Corrected.

- line 106 &ldquo;were broadest the two Ceratiocaris species&rdquo; lacks a work, for the two? In the two?

As the results have slightly changed with the re-orientation of the carapaces, this sentence has been removed.

- Figures 4 &amp; 5, for clearer visualization, would it be possible to use a color that is not part of the white-to-blue magnitude scale for the carapaces themselves (e.g. red or green), so that the highest pressures and velocities are more easily seen?

The carapaces are now shown in purple, which makes the white-blue transition easier to see. Thank you for the suggestion. Green and red clashed too much with the blue and made it (to our eyes) also difficult to see the white-blue transition, hence why we chose purple.

- lines 134&ndash;135 &ldquo;These results indicate that the carapace was not well adapted for a pelagic mode of life.&rdquo; It would be nice, for the neophyte, to explain a bit more why

This part of the discussion has been expanded, to cover this point in more detail.

- Fig. 6, it would be nice to find a way to identify the different taxa, I guess you could give each point a letter and indicate the taxon name corresponding to each letter in the figure caption.

This is a good suggestion. We have provided labels for each of the points to allow them to be identified in the caption.

- lines 158&ndash;160, the sentence &ldquo;A functional comparison can be made to the elongate posterior spines are known in a likely pelagic Cambrian stem-group arthropod Isoxys longissimus.&rdquo; reads weird, I guess a word is lacking or one must be replaced (or maybe &ldquo;are&rdquo; needs to be dropped?)

We have adjusted this sentence so that it reads correctly.

- line 310 &ldquo;files(25)&rdquo; lacks a space

A space has been added.

- lines 313 &amp; 318 &ldquo;Arenosicaris inflata (a) Caryocaris acoitensis&rdquo; lacks a come after (a)

A comma has been added.

- lines 314 &amp; 319, drop the s for &ldquo;0 degrees&rdquo;

This caption has been adjusted.


Finally, I want to really commend the authors for sharing their data and scripts! I have run the scripts and confirm that everything works. Nevertheless, the way OSF.io works requires renaming correctly the download .zip folders so that they match what&rsquo;s needed for the R scripts. Please add comments to the R scripts to specify how the users need to save the downloaded data to make sure they get it working more easily. It is only about folder naming for script &ldquo;Plottingresults_Caryocaridids.R&rdquo;, but it&rsquo;s much less straightforward for the other script &ldquo;Outline_conversion.R&rdquo;: the setwd path is not the same as the one for the other script (quite confusing) and I had to change line &ldquo;CaryoCharacters&lt;-as.vector(c(&#39;jpg&#39;,&#39;number&#39;,&#39;genus&#39;,&#39;species&#39;))&rdquo; into &ldquo;CaryoCharacters&lt;-as.vector(c(&#39;number&#39;,&#39;genus&#39;,&#39;species&#39;))&rdquo;, and line &ldquo;lf &lt;- list.files(&#39;JPG_noshortpostspines&#39;, full.names = TRUE)&rdquo; into &ldquo;lf &lt;- list.files(, full.names = TRUE)&rdquo; to get it working. Please comment the script in a more straightforward way for users who may not be &ldquo;fluent&rdquo; in R.

Thank you for checking the R code thoroughly. We have added additional comments to the code, indicating specifically how subfolders should be named for it to work correctly. The lines indicated in the Outline_conversion.R file that did not work until adjusted are interesting. On our computer and downloading the files from the OSF, the script worked both with and without the ‘jpg’. A comment has been added to the script to note that this issue might crop up for other users.
