# Supplementary material for: Hydrodynamic performance of Ordovician archaeostracan carapaces
Source: PLoS One. 2024 May 31;19(5):e0304559. doi: 10.1371/journal.pone.0304559 (PMC11142683; doi:10.1371/journal.pone.0304559)
Supplement: Attachment — Submitted filename: PatesXue_R2_ResponseToReviews.docx [file pone.0304559.s007.docx]

PONE-D-23-26410R1

Hydrodynamic performance of Ordovician archaeostracan carapaces

PLOS ONE

Dear Dr. Pates,

Thank you for submitting your manuscript to PLOS ONE. After careful consideration, we feel that it has merit but does not fully meet PLOS ONE’s publication criteria as it currently stands. Therefore, we invite you to submit a revised version of the manuscript that addresses the points raised during the review process.

Please submit your revised manuscript by Jun 10 2024 11:59PM. If you will need more time than this to complete your revisions, please reply to this message or contact the journal office at [plosone@plos.org](mailto:plosone@plos.org). When you're ready to submit your revision, log on to [https://www.editorialmanager.com/pone/](https://eur03.safelinks.protection.outlook.com/?url=https%3A%2F%2Fwww.editorialmanager.com%2Fpone%2F&data=05%7C02%7Cs.pates%40exeter.ac.uk%7Cfa088ee8e9c8435fd46608dc6769dfb4%7C912a5d77fb984eeeaf321334d8f04a53%7C0%7C0%7C638498951634095210%7CUnknown%7CTWFpbGZsb3d8eyJWIjoiMC4wLjAwMDAiLCJQIjoiV2luMzIiLCJBTiI6Ik1haWwiLCJXVCI6Mn0%3D%7C0%7C%7C%7C&sdata=V0nIJWUa2rSCobYstULlEJjhO6f7urVcN0JauhFwy2k%3D&reserved=0) and select the 'Submissions Needing Revision' folder to locate your manuscript file.

Please include the following items when submitting your revised manuscript:

- A rebuttal letter that responds to each point raised by the academic editor and reviewer(s). You should upload this letter as a separate file labeled 'Response to Reviewers'.
- A marked-up copy of your manuscript that highlights changes made to the original version. You should upload this as a separate file labeled 'Revised Manuscript with Track Changes'.
- An unmarked version of your revised paper without tracked changes. You should upload this as a separate file labeled 'Manuscript'.

If you would like to make changes to your financial disclosure, please include your updated statement in your cover letter. Guidelines for resubmitting your figure files are available below the reviewer comments at the end of this letter.

If applicable, we recommend that you deposit your laboratory protocols in [protocols.io](https://eur03.safelinks.protection.outlook.com/?url=http%3A%2F%2Fprotocols.io%2F&data=05%7C02%7Cs.pates%40exeter.ac.uk%7Cfa088ee8e9c8435fd46608dc6769dfb4%7C912a5d77fb984eeeaf321334d8f04a53%7C0%7C0%7C638498951634104658%7CUnknown%7CTWFpbGZsb3d8eyJWIjoiMC4wLjAwMDAiLCJQIjoiV2luMzIiLCJBTiI6Ik1haWwiLCJXVCI6Mn0%3D%7C0%7C%7C%7C&sdata=YzVZI7FDQQykqAXdYVJhU%2Fly0Xo6TBtwrIXSWudFmb8%3D&reserved=0) to enhance the reproducibility of your results. Protocols.io assigns your protocol its own identifier (DOI) so that it can be cited independently in the future. For instructions see: [https://journals.plos.org/plosone/s/submission-guidelines#loc-laboratory-protocols](https://eur03.safelinks.protection.outlook.com/?url=https%3A%2F%2Fjournals.plos.org%2Fplosone%2Fs%2Fsubmission-guidelines%23loc-laboratory-protocols&data=05%7C02%7Cs.pates%40exeter.ac.uk%7Cfa088ee8e9c8435fd46608dc6769dfb4%7C912a5d77fb984eeeaf321334d8f04a53%7C0%7C0%7C638498951634108539%7CUnknown%7CTWFpbGZsb3d8eyJWIjoiMC4wLjAwMDAiLCJQIjoiV2luMzIiLCJBTiI6Ik1haWwiLCJXVCI6Mn0%3D%7C0%7C%7C%7C&sdata=DeG7ltkHMDiuTcTppafDeRa5UHZdsBrjz3zdyRAzYf0%3D&reserved=0). Additionally, PLOS ONE offers an option for publishing peer-reviewed Lab Protocol articles, which describe protocols hosted on [protocols.io](https://eur03.safelinks.protection.outlook.com/?url=http%3A%2F%2Fprotocols.io%2F&data=05%7C02%7Cs.pates%40exeter.ac.uk%7Cfa088ee8e9c8435fd46608dc6769dfb4%7C912a5d77fb984eeeaf321334d8f04a53%7C0%7C0%7C638498951634112547%7CUnknown%7CTWFpbGZsb3d8eyJWIjoiMC4wLjAwMDAiLCJQIjoiV2luMzIiLCJBTiI6Ik1haWwiLCJXVCI6Mn0%3D%7C0%7C%7C%7C&sdata=6cVdgNzdTUC%2FdiNm9%2F5yWzhSOMdriSqlNmEN70kB%2BNg%3D&reserved=0). Read more information on sharing protocols at [https://plos.org/protocols?utm_medium=editorial-email&utm_source=authorletters&utm_campaign=protocols](https://eur03.safelinks.protection.outlook.com/?url=https%3A%2F%2Fplos.org%2Fprotocols%3Futm_medium%3Deditorial-email%26utm_source%3Dauthorletters%26utm_campaign%3Dprotocols&data=05%7C02%7Cs.pates%40exeter.ac.uk%7Cfa088ee8e9c8435fd46608dc6769dfb4%7C912a5d77fb984eeeaf321334d8f04a53%7C0%7C0%7C638498951634116399%7CUnknown%7CTWFpbGZsb3d8eyJWIjoiMC4wLjAwMDAiLCJQIjoiV2luMzIiLCJBTiI6Ik1haWwiLCJXVCI6Mn0%3D%7C0%7C%7C%7C&sdata=qcQfV37vaImnVQhj7eJBltyuDl1OVh18EhpC%2FgO%2FU7g%3D&reserved=0).

We look forward to receiving your revised manuscript.

Kind regards,

Shuan-Hong Zhang, PhD
Academic Editor
PLOS ONE

Journal Requirements:

Please review your reference list to ensure that it is complete and correct. If you have cited papers that have been retracted, please include the rationale for doing so in the manuscript text, or remove these references and replace them with relevant current references. Any changes to the reference list should be mentioned in the rebuttal letter that accompanies your revised manuscript. If you need to cite a retracted article, indicate the article’s retracted status in the References list and also include a citation and full reference for the retraction notice.

Additional Editor Comments:

Dear Dr. Stephen Pates,
Thank you very much for your patience in waiting for reviewer’s comments for a long time. I have now received a constructive and helpful review from a new expert on your revised manuscript. Based on reviewer’s comments and my evaluation of your manuscript, I recommend reconsideration of your manuscript following minor revisions. Please follow the comments and suggestions from reviewer and revise your manuscript carefully.
I am looking forward to receiving the revised version of your manuscript in the near future.
Sincerely,
Shuan-Hong Zhang

We thank the editor for their hard work on the manuscript, and for finding helpful reviewers who have improved the study. Following our changes from comments by the third reviewer, we feel that this study is now ready for publication.

The third reviewer confirmed that we have made the necessary changes following the first two reviews, and provided additional helpful comments. We have taken all this reviewers’ comments into account, and made changes to the relevant parts of the text (see point by point responses below).

[Note: HTML markup is below. Please do not edit.]

Reviewers' comments:

Reviewer's Responses to Questions

**Comments to the Author**

1. If the authors have adequately addressed your comments raised in a previous round of review and you feel that this manuscript is now acceptable for publication, you may indicate that here to bypass the “Comments to the Author” section, enter your conflict of interest statement in the “Confidential to Editor” section, and submit your "Accept" recommendation.

Reviewer #3: All comments have been addressed

2. Is the manuscript technically sound, and do the data support the conclusions?

The manuscript must describe a technically sound piece of scientific research with data that supports the conclusions. Experiments must have been conducted rigorously, with appropriate controls, replication, and sample sizes. The conclusions must be drawn appropriately based on the data presented.

Reviewer #3: Yes

3. Has the statistical analysis been performed appropriately and rigorously?

Reviewer #3: Yes

4. Have the authors made all data underlying the findings in their manuscript fully available?

The [PLOS Data policy](https://eur03.safelinks.protection.outlook.com/?url=http%3A%2F%2Fwww.plosone.org%2Fstatic%2Fpolicies.action%23sharing&data=05%7C02%7Cs.pates%40exeter.ac.uk%7Cfa088ee8e9c8435fd46608dc6769dfb4%7C912a5d77fb984eeeaf321334d8f04a53%7C0%7C0%7C638498951634120293%7CUnknown%7CTWFpbGZsb3d8eyJWIjoiMC4wLjAwMDAiLCJQIjoiV2luMzIiLCJBTiI6Ik1haWwiLCJXVCI6Mn0%3D%7C0%7C%7C%7C&sdata=hbH3%2BlADc46QOV0J8e9PRnFNaA5B4ZxntwhpdtjB6L0%3D&reserved=0) requires authors to make all data underlying the findings described in their manuscript fully available without restriction, with rare exception (please refer to the Data Availability Statement in the manuscript PDF file). The data should be provided as part of the manuscript or its supporting information, or deposited to a public repository. For example, in addition to summary statistics, the data points behind means, medians and variance measures should be available. If there are restrictions on publicly sharing data—e.g. participant privacy or use of data from a third party—those must be specified.

Reviewer #3: Yes

5. Is the manuscript presented in an intelligible fashion and written in standard English?

PLOS ONE does not copyedit accepted manuscripts, so the language in submitted articles must be clear, correct, and unambiguous. Any typographical or grammatical errors should be corrected at revision, so please note any specific errors here.

Reviewer #3: Yes

6. Review Comments to the Author

Please use the space provided to explain your answers to the questions above. You may also include additional comments for the author, including concerns about dual publication, research ethics, or publication ethics. (Please upload your review as an attachment if it exceeds 20,000 characters)

Reviewer #3: Review: Hydrodynamic performance of Ordovician archaeostracan carapaces

General Comments

Pates and Xue provide an interesting article about the hydrodynamic properties of Ordovician archaeostracans. The article is sound and aligns with the standards of the field. The article has already been reviewed by two independent researchers besides this review. I believe that the authors have succesfully identified the main problems of their study and addressed them. I recommend publication after this round of review.

Evaluation of the comments addressed to previous reviewers

Reviewer 1: Reviewer 1 brings good points regarding data accuracy and about the sources of the outlines, as well as other helpful comments, which I think have adequately been corrected by the authors. Here, I review some of their main points.

1) The main problem highlighted by Reviewer 1 is the use of published 2D outlines from the literatures.

The authors have provided a new section (Taxonomic sampling) reviewing all outlines used and their origins, which in my opinion, would suffice. Regarding the use of 2D outlines, from my perspective:

Using outlines from the literature is a standard practice in geometric morphometric analyses of palaeozoic invertebrates, especially when outlines are as simple as carapace valves (e.g., Caron & Moysiuk, 2021RSOS). Ideally, the authors would have examined the material themselves using state-of-the-art imaging. However, this requires an important investment in time and economic resources, including multiple international trips, access to different collections (...) to acquire a complete dataset.

Using previously described outlines is an adequate solution to this problem in tandem with a careful examination of any other type of evidence (most commonly, images provided by the different institutions or included in their respective publications). Using images alone from previous publications can also introduce biases, as their quality in older publications is often low. Although new technologies (e.g., cross-polarizing photography) have improved our examination of fossil material, past researchers often excelled at diagrammatic reconstructions, and, unless there are major differences between their reconstructions and the images available, these reconstructions can normally be used as reliable evidence.

The study focuses on interspecific differences and thus, even if there are small changes between the real carapace shape and the reconstruction/image, these differences do not impact heavily interspecific differences, as the authors comment in their main text.

2) Wrong reconstructions for J. raymondi and C. angusta.

Reviewer 1 correctly identifies this issue. The authors have correctly added a note in their main text. Given that these species are not relevant in the discussion, I believe is adequate to include them on account of including as many Ordovician archeostracans as possible.

3) 3D analyses.

I agree with the authors that a 3D analysis, while interesting, would also introduce additional variables to consider. Carapace valves are usually found in isolation and two-dimensionally compressed. 3D analyses of other palaeozoic arthropods like trilobites can only be easily peformed because they are, in nature, three-dimensional fossils. In order to perform a 3D analysis, the authors must reconstruct two main features: the attachment between both valves (i.e., the closure of the carapace) as well as the three-dimensionality of the carapace valve (i.e., whether it is bulging or flat). These two factors could be extrapolated using multiple specimens of the same species showing compression artefacts and different orientations of the carapace (in different views, for example), and taking into account the mode of preservation. This is already quite difficult for a single species, for which this material may not be present.

Furthermore, as the authors point out, in order to create a hydrodynamic model, a 2D shape is suitable. To my knowledge, there is no specific criteria data must follow to create an hydrodynamic model. There have already been similar studies using the same methods and 2D shapes (e.g., Pates et al., 2021 ProcB; Meyers & Msomi, 2021, Materialstoday) even in engineering journals (e.g., Mohamed et al., 2021. J.W.E.I.A). Of course, the more data available, the more accurate the hydrodynamic model is, including three-dimensional models, effect of the legs and tail, size, type of cuticle... These features may be easily accessible for extant taxa, but are difficult to obtain in the fossil record, and thus, a simple 2D approach is appropriate as long as the discussion and conclusion focus on broad patterns.

Reviewer 2: Reviewer 2 brings valuable comments that, in my opinion, have correctly been assessed by the authors. The main issue brought by the reviewer was the incorret orientation of two species of the dataset, which has been corrected accordingly.

We thank the reviewer for their careful assessment of the previous reviewers’ comments and our changes in response.

Additional reviews

I think the abstract does not completely capture the results of the paper. To me, the main points are that there is a general trend across archeostracan evolution towards lowering drag and improving lift, which is quite clear in the origin of caryocaridids, which could be related to their change into a pelagic lifestyle. It is good to acknowlege further adaptations, but the importance of the appendages or thinning of the carapace are not widely covered in the main text. I think at least a mention to drag and lift is necessary, and I encourage the authors to decide on their best way to highlight their other results.

Smaller comments
34- delete extra space between of and arthropods deleted, thank you

39- can the authors provide a reference or reconsider the word "zoonekton"? I don't think this a very common term; a quick search reveals almost no previous references using it. We have removed this word from the text, instead referring to ‘*macroscopic pelagic animals’*.

197- what does "wake" mean in this sentence? It refers to the flow behind the carapace that has low velocity – i.e. the area that has been affected by the carapace. We have rephrased this sentence to avoid the use of this word, as it was unknown to the reviewer. This sentence now reads ‘*Visualisation of the velocity contours for the same carapaces reveals a broader and more elongate area of low velocity flow posterior to the carapace…’*

210- change "a" to "at" added ‘at’

210-211- I think that the sentence "taxa at a higher number of nodes" should be changed. Although I understand what the authors mean, it is not very accurate, as the number of nodes depend on the number of diversification events across the tree. I think what the authors mean is that in general, caryocaridids, have lower drag coefficient and higher lift:drag ratio than ceratiocarids, which also have lower drag and higher lift than Aresinocaris.

The number of nodes from *Arenosicaris inflata* was used as a coarse proxy for tree path distance. We appreciate that it does depend on the diversification events in the tree (and in the case of the tree used here, the number of polytomies will influence the number of nodes). We have rephrased this paragraph to make this clearer.

*‘Plots of minimum drag coefficient against highest lift:drag ratio demonstrate a likely relationship between these two metrics of hydrodynamic performance (Fig 6). Carapaces of taxa at a greater tree path distance (number of nodes used as a proxy) from Arenosicaris inflata (Fig 1) are broadly more hydrodynamic (higher lift:drag ratio, lower minimum drag coefficient) than those fewer nodes away.’*

213- please specify the correlation, as the reader may think is between drag and lift. I think what the authors mean is that there may be a trend towards lower drag and higher lift towards the origin of caryocaridids but cannot test it with (e.g.) phylogenetic tracing.

We now specify the correlation. The text now reads: *‘The sample size is too small to statistically test for correlation between tree path distance from A. inflata (number of nodes used as a proxy) and highest lift:drag ratio, or minimum drag coefficient.’*

233- Ce. angusta (italics) corrected, thank you

235- delete ";" after lethiersi corrected, thank you

241- Thus, added comma, thank you.

260- Again, the use of "the node above" is a little bit strange from a phylogenetic perspective. In this case, for example, I think it is easier to state that the morphology probably appeared at the common ancestor of all Ordovician archeostracans, and was secondarily lost in Pumilocaris. This phrasing is better than our original, and we have rephrased as suggested.

275-better state "This suggests that for this stem-group arthropod without apparent appendage specialization and without abdomen, adaptations of the carapace played a more important carapace role". This is because "were not possible" assumes that their stem-group position limits their potential morphological adaptations. This is not something we can easily assume, especially when similar taxa like Occacaris have an abdomen and that new information is increasingly revealing new adaptations regarding the legs of Isoxys (e.g., the endites recorvered by Zhang et al., 2021). We agree that our original phrasing was not appropriate. We have rewritten this part, which now reads *‘This suggests that for a stem-group arthropod without apparent appendage specialisation and without an abdomen, adaptation of the carapace may have played a more important role.’*

280- Maybe the authors could separate this into different sentences, as it is a little bit unclear to me when written as one singular sentence. By elongating the body, do the authors mean that the animal would stretch its abdomen? If that is the case, how does it change the separation of the flow? Would swimming speed necessary to achieve flow separation be reduced, then? Is there any citation or further evidence (e.g, carapace shape) the authors could use to back up this claim?

We have removed this section, where references to flow separation were made. This paragraph is now greatly shortened, and reads:

*‘For archaeostracans there is no evidence that the abdomen could dorsally flex (and so a ‘power stroke’ as in Nebalia may not have been a major way of providing thrust [22]), however the abdomen may have still been important for escape reactions [22]. In addition to carapace morphology and the abdomen, thrust provided by specialised swimming appendages were likely also important for facilitating a pelagic mode of life in the group [20,22].’*

References: I have similarly experienced problems with Mendeley's citations. My recommendation to the authors is to edit the PLOS style in Mendeley under a different name and make sure to always use the same account and preferably, device. Mendeley often fails to update properly across different devices, so changing the document from one to another device can lead to errors.

Thank you for the advice and for the suggestions below. We have corrected the references as indicated.

323: bioturbation: an updated account.
325: Isoxys (italics)
328: ecosystem: evidence
333: zooplankton: bradoriid
342: (GOBE): the palaeocological
347: trilobites: the invasion
355: Microparia speciosa (italics=
387: Dimorphism of bivalved arthropod
405: Momocs: outline analysis
407: Swimming soeed and oxygen consupmtion in the bathypelagic mysid
416: Origins, evolution and diversification of zooplankton.

7. PLOS authors have the option to publish the peer review history of their article ([what does this mean?](https://eur03.safelinks.protection.outlook.com/?url=https%3A%2F%2Fjournals.plos.org%2Fplosone%2Fs%2Feditorial-and-peer-review-process%23loc-peer-review-history&data=05%7C02%7Cs.pates%40exeter.ac.uk%7Cfa088ee8e9c8435fd46608dc6769dfb4%7C912a5d77fb984eeeaf321334d8f04a53%7C0%7C0%7C638498951634124063%7CUnknown%7CTWFpbGZsb3d8eyJWIjoiMC4wLjAwMDAiLCJQIjoiV2luMzIiLCJBTiI6Ik1haWwiLCJXVCI6Mn0%3D%7C0%7C%7C%7C&sdata=bM1ysDTk7b1mXINVbLGbp6PbMIE2GjFxyo%2BRw3jCsrA%3D&reserved=0)). If published, this will include your full peer review and any attached files.


If you choose “no”, your identity will remain anonymous but your review may still be made public.


**Do you want your identity to be public for this peer review?** For information about this choice, including consent withdrawal, please see our [Privacy Policy](https://eur03.safelinks.protection.outlook.com/?url=https%3A%2F%2Fwww.plos.org%2Fprivacy-policy&data=05%7C02%7Cs.pates%40exeter.ac.uk%7Cfa088ee8e9c8435fd46608dc6769dfb4%7C912a5d77fb984eeeaf321334d8f04a53%7C0%7C0%7C638498951634127998%7CUnknown%7CTWFpbGZsb3d8eyJWIjoiMC4wLjAwMDAiLCJQIjoiV2luMzIiLCJBTiI6Ik1haWwiLCJXVCI6Mn0%3D%7C0%7C%7C%7C&sdata=G8Y8Tw1GvuzspPnJKIULJbZMi%2BhA%2BlYEyGOYO8%2FqEOA%3D&reserved=0).

Reviewer #3: No

[NOTE: If reviewer comments were submitted as an attachment file, they will be attached to this email and accessible via the submission site. Please log into your account, locate the manuscript record, and check for the action link "View Attachments". If this link does not appear, there are no attachment files.]

While revising your submission, please upload your figure files to the Preflight Analysis and Conversion Engine (PACE) digital diagnostic tool, [https://pacev2.apexcovantage.com/](https://eur03.safelinks.protection.outlook.com/?url=https%3A%2F%2Fpacev2.apexcovantage.com%2F&data=05%7C02%7Cs.pates%40exeter.ac.uk%7Cfa088ee8e9c8435fd46608dc6769dfb4%7C912a5d77fb984eeeaf321334d8f04a53%7C0%7C0%7C638498951634131711%7CUnknown%7CTWFpbGZsb3d8eyJWIjoiMC4wLjAwMDAiLCJQIjoiV2luMzIiLCJBTiI6Ik1haWwiLCJXVCI6Mn0%3D%7C0%7C%7C%7C&sdata=wVRqjeb4hJ1DHZkMZNtZ8%2B87Mf%2FYQOBIq2OyJY%2FZUoM%3D&reserved=0). PACE helps ensure that figures meet PLOS requirements. To use PACE, you must first register as a user. Registration is free. Then, login and navigate to the UPLOAD tab, where you will find detailed instructions on how to use the tool. If you encounter any issues or have any questions when using PACE, please email PLOS at [figures@plos.org](mailto:figures@plos.org). Please note that Supporting Information files do not need this step.
